# Outside-in integrin signalling regulates haematopoietic stem cell function via Periostin-Itgav axis

**DOI:** 10.1038/ncomms13500

**Published:** 2016-12-01

**Authors:** Satish Khurana, Sarah Schouteden, Javed K. Manesia, Albert Santamaria-Martínez, Joerg Huelsken, Adam Lacy-Hulbert, Catherine M. Verfaillie

**Affiliations:** 1 Inter-departmental Stem Cell Institute, KU Leuven, 3000 Leuven, Belgium; 2 École Polytechnique Fédérale de Lausanne (EPFL), 1015 Lausanne, Switzerland; 3 Benaroya Research Institute at Virginia Mason, Seattle, WA 98101, USA

## Abstract

Integrins play an important role in haematopoietic stem cell (HSC) maintenance in the bone marrow niche. Here, we demonstrate that Periostin (Postn) via interaction with Integrin-αv (Itgav) regulates HSC proliferation. Systemic deletion of Postn results in peripheral blood (PB) anaemia, myelomonocytosis and lymphopenia, while the number of phenotypic HSCs increases in the bone marrow. *Postn*^*−/−*^ mice recover faster from radiation injury with concomitant loss of primitive HSCs. HSCs from *Postn*^*−/−*^ mice show accumulation of DNA damage generally associated with aged HSCs. Itgav deletion in the haematopoietic system leads to a similar PB phenotype and HSC-intrinsic repopulation defects. Unaffected by Postn, *Vav-Itgav*^*−/−*^ HSCs proliferate faster *in vitro*, illustrating the importance of Postn-Itgav interaction. Finally, the Postn-Itgav interaction inhibits the FAK/PI3K/AKT pathway in HSCs, leading to increase in p27Kip1 expression resulting in improved maintenance of quiescent HSCs. Together, we demonstrate a role for Itgav-mediated outside-in signalling in regulation of HSC proliferation and stemness.

## Abstract (web summary)

Integrins regulate haematopoietic stem cell (HSC) homeostasis and engraftment into the bone marrow (BM) niche upon transplantation. Here, the authors show that HSC quiescence and function in the BM is regulated by the interaction of PERIOSTIN and INTEGRIN αv and subsequent increase in p27Kip1.

## Graphical abstract

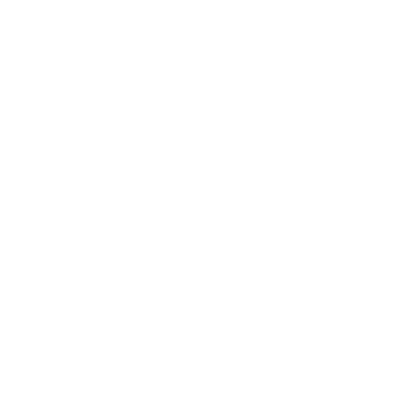

Haematopoietic stem cells (HSCs) respond to signals from their niche through a repertoire of cell surface receptors, of which integrins constitute a very important class^1^. Integrins not only aid in cell-extracellular matrix (ECM) and cell–cell adhesion, but are also known to transduce extracellular signals that affect cell fate^2^. Integrins are a class of heterodimeric trans-membrane receptors composed of an α- and a β-chain, consisting of a large extracellular domain, a single pass trans-membrane domain and a smaller intracellular domain^3^. Integrins bind to ECM molecules in addition to a variety of cytokines, growth factors, proteases and proteins expressed on the surface of adjacent cells^4^. HSCs express a variety of integrins, which play important roles in their maintenance within the bone marrow (BM) niche, regulate their egress from the niche, and other functions^5^,^6^. For instance, neutralization of VLA4 (α4β1 heterodimer) by blocking antibodies prevent their *in vitro* attachment to ECM and spleen colony formation *in vivo*^7^,^8^.

Among other integrins, it has been shown that primitive HSCs from murine BM express the integrin-β3 (ref. 9). Thrombopoietin (TPO)-mediated HSC maintenance depends on inside-out signalling through activation of the integrin-αvβ3 (refs 10, 11). Integrin-αvβ3 also plays an important role in vasculogenesis during embryogenesis and tumour development^12^. Apart from binding vitronectin, Integrin-αvβ3 interacts with other ECM proteins, such as fibrinogen, fibronectin and thrombospondin; growth factors, such as platelet-derived growth factor, insulin and vascular endothelial growth factor receptor-2; and to the protease matrix metalloproteinase^13^. In addition, Integrin-αvβ3 expression was found to be essential in maintenance of leukaemic stem cells in the *MLL-AF9* mouse model^14^.

Another ECM molecule, Periostin (Postn), also binds to αvβ3 and αvβ5 integrins^15^ and can induce outside-in signalling via activation of focal adhesion kinase (FAK)^16^. Postn plays an important role in the development of heart and is involved in many of its pathologies^17^. Moreover, Postn has been shown to mediate smooth muscle cell migration by FAK mediated signalling via integrins αvβ3 and αvβ5 (ref. 18). Initially identified in a mouse osteoblastic cell line^19^, Postn is expressed in many cell types, and has more recently been found in multiple cancer tissues such as breast cancer^20^, lung cancer^21^, colon cancer^22^, pancreatic cancer^23^ and ovarian cancer^24^ among others^25^.

Various mechanisms that regulate proliferation have been shown to affect HSC stemness^26^. Aside from cytokines and growth factors, engagement of integrins, such as binding of VLA4 to vascular cell adhesion molecule and fibronectin, affects HSC proliferation^27^. Here, we demonstrate that Postn regulates HSC proliferation by direct interaction with Itgav. This interaction results in increased expression of *p27Kip1* (*Cdkn1b*) caused by Postn-mediated inhibition of the FAK/PI3K/Akt pathway, which has been shown to induce quiescence of HSCs (ref. 28). In *Postn*^*−/−*^ mice, we observed increased proliferation of haematopoietic stem and progenitor cells (HSPCs) combined with faster functional decline of HSCs following hematopoietic injury, as well as skewing of haematopoiesis in older *Postn*^*−/−*^ mice, which has previously been suggested to be a sign of replication stress^29^. Likewise, we demonstrate that short-term as well as long-term engraftment of HSCs from *Itgav*^*fl/fl*^*;Vav-icre* mice is decreased, and we also found skewed haematopoietic output of HSCs in these mice. Consistent with recent studies^29^, our results implicate replication stress in the functional decline of HSCs.

## Results

### Postn inhibits culture-induced proliferation of BM HSCs

**Figure 1:**
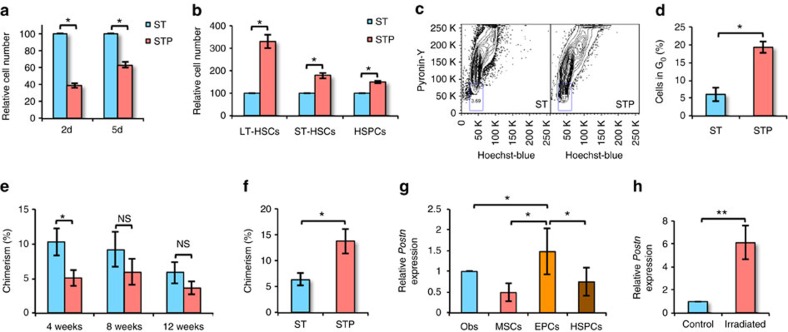
Postn inhibits culture-induced proliferation of BM HSCs. (**a**) Sorted KLS cells cultured in serum-free medium with SCF+TPO in the presence (STP) or absence (ST) of Postn were harvested after 2 or 5 days and total cell expansion was compared (*n*=6, *t* test: *P<0.001). (**b**) Cells harvested after 5 days of culture were analysed for HSPC sub-populations by flowcytometry. Relative proportions of the sub-populations are plotted (*n*=5, *t* test: *P<0.005). (**c**) Cell cycle analysis of the harvested cells by Hoechst 33342/pyronin Y staining (*n*=6). (**d**) Comparison of the proportion of harvested cells, in G_0_ of cell cycle (*n*=6, *t* test: *P=0.002). (**e**) PB chimerism in primary recipients lethally irradiated and transplanted with the progeny of 200 KLS cells cultured with or without Postn along with 100,000 competitor BM cells (*n*=3, *N*=12, *t* test: *P=0.037). (**f**) PB chimerism in secondary recipients lethally irradiated and transplanted with 1 × 10^6^ BM cells from primary recipients that received KLS cells cultured with/without Postn (*n*=3, *N*=12, *t* test: *P=0.015). (**g**) Expression of *Postn* in different sub-population sorted from the total BM cells isolated from WT mice (*n*=5, *t* test: *P<0.05). (**h**) Expression of *Postn* in non-hematopoietic (Lin^−^CD45^−^) BM cells magnetic-activated cell sorting (MACS) sorted from the total BM cells isolated from control or lethally irradiated (24 h) WT mice (*n*=4, *t* test: **P=0.028). (*n*=independent experiments, *N*=number of mice. Error bars indicate mean ±s.e.m.).

We cultured BM derived Lin^−^Sca-1^+^c-kit^+^ (KLS) cells in serum-free medium containing stem cell factor (SCF) and TPO with or without Postn for up to 5 days. As reported in earlier studies^30^, KLS cells cultured with SCF/TPO lose their quiescence and start proliferating. We observed a decrease in the expansion of KLS cells cultured in the presence of Postn (≥2 mg ml^−1^) within 2 days (Fig. 1a, Supplementary Fig. 1A,B). The number of cells harvested after 5 days of culture was enumerated (Supplementary Fig. 1B) and the proportion of phenotypically defined HSC subpopulations was examined by flowcytometry. We observed an increase in the proportion (Fig. 1b) as well as absolute number (Supplementary Fig. 1C) of HSPCs (haematopoietic stem and progenitors; c-Kit^+^Lin^−^Sca1^+^ or KLS cells), short-term (ST-)HSCs (CD150^−^CD48^−^ KLS cells) and long-term (LT-)-HSCs (CD150^+^CD48^−^ KLS or SLAM KLS cells). Differences in the cell number could not be attributed to changes in apoptosis as there was no change in Annexin V^+^ HSCs following culture with/without Postn (Supplementary Fig. 1D). Consistent with the increased proportion of KLS cells, methylcellulose colony-forming unit assays demonstrated that the number of colony-forming unit granulocyte, erythroid, monocyte and megakaryocyte was higher in cultures with Postn (Supplementary Fig. 1E). Using Hoechst 33342 (Ho) staining we found that culture in the presence of Postn resulted in a decreased fraction of cells in G_2_/M phase of the cell cycle (Supplementary Fig. 1F), while staining with a combination of Hoechst 33342/Pyronin Y (Ho/Py; Fig. 1c) identified a greater proportion of KLS cell progeny from Postn containing cultures to be in the G_0_ stage of the cell cycle (Fig. 1d). We also examined cell cycle status of the KLS cell fraction within the cells harvested following culture (Supplementary Fig. 1G). Although there was a decrease in the proportion of cells in G_0_/G_1_ stage and increase in the cells in S and G_2_/M stage of the cell cycle, the differences were modest compared with the total cells, suggesting that the cell cycle status of the stem cell population did not change much, while the increase in the stem cell fraction in harvested cells was more pronounced.

We next performed competitive repopulation assays to assess the functional properties of cells cultured with Postn compared with control (Fig. 1e,f). Progeny of 200 CD45.1 KLS cells, cultured with/without Postn was transplanted along with 100,000 CD45.2 whole BM cells (WBMCs) in lethally irradiated CD45.2 animals. Peripheral blood (PB) chimerism was analysed every 4 weeks. We also performed secondary transplantations by grafting 100,000 WBMCs harvested from primary recipients after 12 weeks of transplantation. At 4 weeks following transplantation, PB chimerism in primary recipients transplanted with Postn cultured KLS progeny was lower than in animals grafted with cells cultured without Postn (Fig. 1e). We could not detect any difference in chimerism between primary recipients that received cells cultured with or without Postn 8 and 12 weeks following transplantation. However, chimerism in secondary recipients 12 weeks after transplantation of WBMCs from animals grafted with Postn cultured cells was higher compared with control (Fig. 1f). In addition, we observed higher B-cell chimerism in secondary recipients that received BM from mice transplanted with Postn cultured cells (Supplementary Fig. 1H). Thus, culture of KLS cells with Postn resulted in decreased HSC proliferation and differentiation, which was associated with an increase in phenotypic and functionally defined long-term repopulating HSCs.

Next, we examined if Postn is expressed in the cellular components of HSC niche in the BM (Fig. 1g). As published before^31^, we sorted osteoblasts (lin^−^CD45^−^Ter119^−^CD51^+^Sca-1^−^), mesenchymal stem cells (lin^−^CD45^−^Ter119^−^CD51^+^Sca-1^+^) and endothelial progenitor cells (lin^−^CD45^−^Ter119^−^CD31^+^Sca-1^+^) to analyse expression of *Postn*. We observed the highest expression of Postn in the endothelial progenitors. We also observed that the expression of *Postn* increased in the endothelial progenitors isolated from the BM of irradiated animals when evaluated 24 h post-irradiation (Fig. 1g). Interestingly, we also found elevated levels of *Postn* transcripts in lin^−^CD45^−^ cell fraction (Fig. 1h). As Postn is a secreted protein, overall levels of the protein in BM plasma, autocrine regulation of HSC function by HSC-derived Postn cannot be ruled out. However, as our *in vitro* studies demonstrated that Postn increased maintenance of HSC stemness, autocrine Postn effects, if existed, may not be sufficient to maintain primitive HSCs *ex vivo*.

### Increased proliferation of HSCs in *Postn* deficient BM

**Figure 2:**
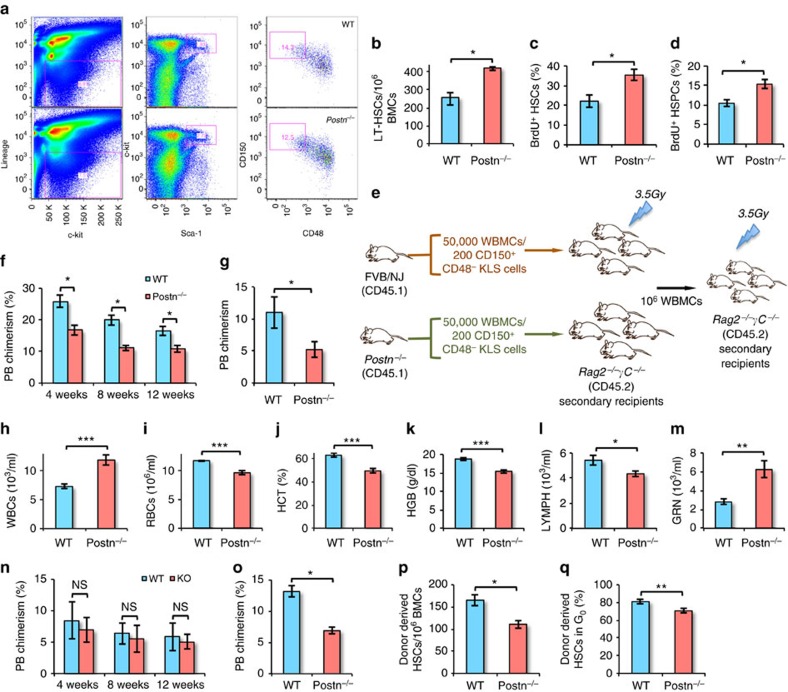
Postn deficiency leads to pre-mature exhaustion of haematopoietic system. (**a**) Flowcytometry analysis of the BM cells derived from 16-week-old FVB/NJ (WT; upper panel) and *Postn*^*−/−*^ (KO; lower panel) mice (*N*=12). (**b**) Frequency of SLAM KLS cells per million BM cells derived from 16-week-old FVB/NJ (WT) and *Postn*^*−/−*^ (KO) mice (*N*=12, *t* test: *P<0.008). (**c**,**d**) BrdU incorporation assays to examine the proliferation status of KLS cells (ST-HSCs; **c**) and SLAM KLS cells (LT-HSCs; **d**) in WT and *Postn*^*−/−*^ mice. BrdU staining in addition to HSC markers in BM cells following 3 (**c**) or 7 (**d**) days of BrdU infusion (*n*=3, *N*=9, *t* test: *P<0.02). (**e**) Schematic representation of the competitive repopulation assays. 50,000 total BM cells derived from WT/*Postn*^*−/−*^ mice (CD45.2) were transplanted into sub-lethally irradiated Rag2^−/−^γC^−/−^ mice. PB chimerism was followed for 12 weeks, after which secondary transplantations were performed. (**f**,**g**) Donor-derived PB chimerism in primary (**f**) and secondary (**g**) recipients transplanted with total BM cells from 8-week-old WT/*Postn*^*−/−*^ mice (*n*=3, *N*=18, *t* test: *P<0.03). (**h**–**m**) Blood obtained from 16-week-old wild-type (WT) and *Postn*^*−/−*^ mice was assessed for WBC count (**h**), RBC count (**i**), haematocrit value (**j**), haemoglobin level (**k**), lymphocyte (**l**) and granulocytes (**m**) numbers (*N*=12, *t* test: ***P<0.001, **P<0.01, *P<0.05). (**n**,**o**) Donor-derived PB chimerism in primary (**n**) and secondary (**o**) recipients transplanted with sorted primitive HSCs (CD150^+^CD48^−^KLS cells) total BM cells from 16-week-old WT/*Postn*^*−/−*^ mice (*n*=3, *N*=18, *t* test: *P=0.02). (**p**) Frequency of primitive HSCs in the donor-derived fraction of BM cells from secondary recipients (*n*=3, *N*=6, *t* test: *P=0.007). (**q**) Proportion of donor-derived primitive HSCs in secondary recipients in G0 stage of cell cycle (*n*=3, *N*=6, *t* test: **P=0.001). (*n*=independent experiments, *N*=number of mice. Error bars indicate mean±s.e.m.).

We next evaluated the haematopoietic potential of HSCs from *Postn*^*−/−*^ mice. Genotyping was performed to identify *Postn*^*−/−*^ animals (Supplementary Fig. 2A) and lack of *Postn* expression in the BM tissue of the *Postn*^*−/−*^ mice was confirmed by quantitative PCR with reverse transcription (qRT-PCR; Supplementary Fig. 2B). BM cells from wild-type (WT) and *Postn*^*−/−*^ mice were analysed for the frequency of LT-HSCs (SLAM KLS cells) per million BM cells by flowcytometry (Fig. 2a,b). We observed a higher frequency of SLAM KLS cells per million WBMCs in *Postn*^*−/−*^ mice compared with WT mice (Fig. 2b), while there was an overall decrease in BM cellularity in *Postn*^*−/−*^ mice (normalized on body weight; Supplementary Fig. 2C). Bromodeoxyuridine (BrdU) incorporation assays demonstrated that an increased proportion of KLS cells were labelled with BrdU in *Postn*^*−/−*^ mice (Fig. 2c), suggesting HSPC proliferation. In these experiments wherein BrdU was injected for 3 days, we could not find any difference in BrdU incorporation by LT-HSCs (Supplementary Fig. 2D). As LT-HSCs proliferate at a slower rate, we repeated these experiments with BrdU injection for 7 days. In these experiments, we observed a clear increase in the proportion of LT-HSCs that incorporated BrdU and hence were proliferating (Fig. 2d, Supplementary Fig. 2E).

To assess the functional properties of HSCs from *Postn*^*−/−*^ mice, we performed competitive repopulation assays (Fig. 2e–g). In the absence of CD45.2 FVB congenic mice, 50,000 WBMCs from 8-week-old WT or *Postn*^*−/−*^ mice were transplanted in sub-lethally (3.5 Gy) irradiated *Rag2*^*−/−*^*γC*^*−/−*^ (CD45.2) mice (Fig. 2e). PB chimerism was analysed every 4 weeks and after 12 weeks, 100,000 WBMCs were transplanted in secondary mice. Although the frequency of SLAM KLS cells was higher in *Postn*^*−/−*^ mice, we observed lower chimerism in primary (*t* test: *P=0.02; Fig. 2f) as well as secondary (Fig. 2g) recipients transplanted with *Postn*^*−/−*^ WBMCs. We also observed a moderate increase in donor chimerism in the myeloid lineage but a decrease in the B- and T-cell lineage in animals grafted with *Postn*^*−/−*^ BM cells (Supplementary Fig. 2F). These results demonstrated that compared with WT HSCs, HSCs in *Postn*^*−/−*^ mice proliferate faster, leading to expansion of the phenotypically defined HSC pool, however, with a decreased repopulation ability and a modest bias towards the myeloid lineage.

### Altered production of mature blood cells in *Postn* ^*−/−*^ mice

We next performed PB analysis of the *Postn*^*−/−*^ mice in comparison with WT mice to assess the ability of HSCs to maintain homeostasis. In 8-week-old *Postn*^*−/−*^ mice (Supplementary Fig. 2G–M), we found a modest decrease in red blood cell count (RBC; Supplementary Fig. 2G) as well as haematocrit values (Supplementary Fig. 2H), but no change in haemoglobin levels (Supplementary Fig. 2I). In addition, *Postn*^*−/−*^ mice had more white blood cells (WBCs; Supplementary Fig. 2J) caused by increase in granulocyte numbers (Supplementary Fig. 2K). No change in the proportion of lymphocytes (Supplementary Fig. 2L) or platelets (Supplementary Fig. 2M) was observed. Interestingly, we observed that the differences in the blood counts between WT and *Postn*^*−/−*^ mice were more pronounced in 16-week-old mice. We detected a greater increase in WBC counts (Fig. 2h) in 16-week-old *Postn*^*−/−*^ mice than in 8-week-old mice. There was a concomitant decrease in the RBC counts (Fig. 2i), haematocrit values (Fig. 2j) and haemoglobin levels (Fig. 2k). In addition, in 16-week-old mice, unlike 8-week-old mice, we found a decrease in lymphocytes in the PB (Fig. 2l) as well as a further increase in granulocyte numbers (Fig. 2m). As in 8-week-old mice, platelet count was not changed even in 16-week-old mice (Supplementary Fig. 2N). Although these results do not present any direct causative link between the lack of Postn and aging, it is noteworthy that aged mice show a similar haematopoietic phenotype, where increased myelopoiesis with decreased lymphopoiesis is observed^32^. Our experiments also showed decreased expression of Postn in the non-haematopoietic fraction of BM of aged mice (Supplementary Fig. 2O). In addition, multiple mouse models with poor HSC function with faster exhaustion have been reported with this phenotype, as reviewed recently^33^.

As the haematopoietic phenotype was more pronounced in 16-week compared with 8-week-old *Postn*^*−/−*^ mice, we repeated long-term repopulation assays using sorted primitive HSCs from 16-week-old mice to further assess their function (Fig. 2n,o). Sub-lethally (3.5 Gy) irradiated *Rag2*^*−/−*^*γC*^*−/−*^ mice were transplanted with 200 primitive HSCs (CD150^+^CD48^−^KLS cells) derived from 16-week-old WT or *Postn*^*−/−*^ mice. We did not observe any difference in donor-derived chimerism in primary recipients transplanted with HSCs from WT or *Postn*^*−/−*^ mice (Fig. 2n). However, donor-derived chimerism in secondary recipients of *Postn*^*−/−*^ HSCs was decreased (Fig. 2o). In addition, we observed a lower frequency of donor-derived primitive HSCs than recipient HSCs (Fig. 2p). Further analysis of the cell cycle status of these donor cells showed a marginal decrease in the proportion of primitive HSCs in G_0_ stage of cell cycle (Fig. 2q, Supplementary Fig. 2P). This suggests poor long-term potential of primitive HSCs derived from 16-week-old *Postn*^*−/−*^ mice.

### *Postn* ^*−/−*^ mice recover faster from haematopoietic injury

**Figure 3:**
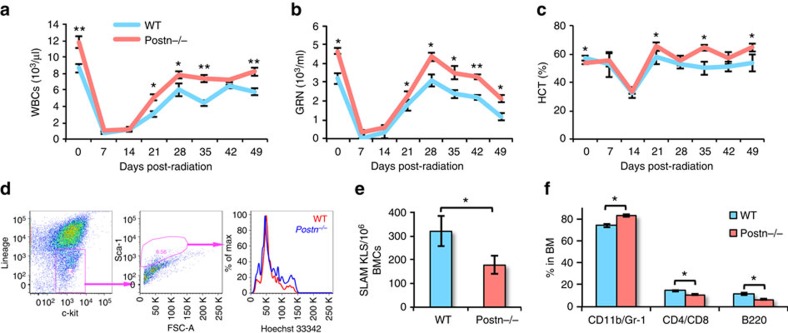
Faster exhaustion of HSCs in *Postn*^*−/−*^ mice following haematopoietic injury. (**a**–**c**) Following sub-lethal irradiation PB counts were measured weekly for 7 weeks. Numbers of WBCs (**a**), granulocytes (**b**), and haematocrit values (**c**) were compared between FVB/NJ (WT) and *Postn*^*−/−*^ (KO) mice (*n*=3, *N*=18, *t* test: **P<0.01, *P<0.05). (**d**) Following sub-lethal irradiation the cell cycle status of BM derived KLS cells was analysed. Two weeks after irradiation, BM cells were isolated and flowcytometry analysis was performed to assess proliferation of lin^−^c-kit^+^Sca-1^+^ (KLS) cells with Hoechst labelling (*n*=3, *N*=9). (**e**,**f**) Flowcytometry based analysis performed during 8 weeks following sub-lethal irradiation to compare the number of SLAM KLS (**e**) and various lineage committed (**f**) cells in the BM of FVB/NJ (WT) and *Postn*^*−/−*^ (KO) mice (*n*=3, *N*=9, *t* test *P<0.05). (*n*=independent experiments, *N*=number of mice. Error bars indicate mean ±s.e.m.).

As our studies suggested poor function of primitive HSCs in *Postn*^−/−^ mice, we hypothesized that this might affect the ability of HSCs to rescue these animals from radiation injury. To assess this, WT and *Postn*^*−/−*^ mice were sub-lethally irradiated and the recovery of the haematopoietic system was followed for 7 weeks by weekly blood cell counts (Fig. 3a–c, Supplementary Fig. 3A–C). A faster recovery of total WBC counts (Fig. 3a), granulocyte counts (Fig. 3b) and haematocrit values (Fig. 3c) were seen at 3 weeks following irradiation in *Postn*^*−/−*^ mice. There was a modest increase in the recovery of platelets (Supplementary Fig. 3A), RBC count (Supplementary Fig. 3B), haemoglobin levels (Supplementary Fig. 3C), but no change in lymphocyte recovery in *Postn*^*−/−*^ mice compared with WT mice (Supplementary Fig. 3D). As improved haematopoietic recovery was seen despite lower haematopoietic potential of HSCs in *Postn*^*−/−*^ mice, we hypothesized that faster haematopoietic recovery might be explained by faster cycling of the haematopoietic progenitors. We therefore analysed the cell cycle status of KLS cells in BM of *Postn*^*−/−*^ and WT mice following sub-lethal irradiation (Fig. 3d). On day 14 after sub-lethal irradiation, we observed more proliferative KLS cells in BM of *Postn*^*−/−*^ compared with WT mice (Fig. 3d). We also assessed the frequency of the different HSPC populations in the BM of *Postn*^*−/−*^ and WT mice 8 weeks after irradiation. Although the number of lin^−^c-kit^+^ (Supplementary Fig. 3E) and KLS cells (Supplementary Fig. 3F) in *Postn*^*−/−*^ BM was not affected, the proportion of SLAM KLS cells (Fig. 3e) was reduced. Analysis of lineage-committed cells in the BM also demonstrated an increased number of myeloid cells (CD11b/Gr-1) with reduced T- (CD4/CD8) and B- (B220) lymphoid cells (Fig. 3f) in *Postn*^*−/−*^ compared with WT mice.

### Postn affects HSC proliferation via binding with Itgav

**Figure 4:**
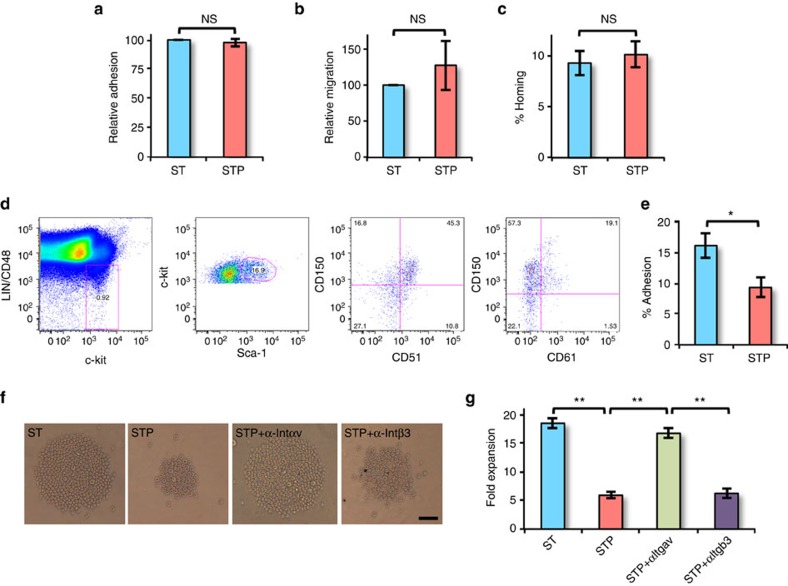
Postn affects HSC proliferation by binding to Itgav. (**a**–**c**) BM derived KLS cells were cultured for 5 days in serum-free medium with SCF and TPO in the absence (ST) or presence (STP) of Postn. (**a**) Cultured BM KLS cells harvested after 5 days were labelled with the PKH-26 dye and plated on ST2 cell feeders. The relative proportion of cells that adhered after 3 h was plotted (*n*=4, *t* test: NS *P*>0.05). (**b**) Migration of cultured BM KLS cell progeny following 5 days of culture, and labelled with the PKH-26 dye, towards SDF-1α was assessed using a trans-well system. The relative proportion of cells that migrated after 3 h was plotted (*n*=4, *t* test: NS *P*>0.05). (**c**) Cultured BM KLS cells harvested after 5 days were tested for their *in vivo* homing capacity, by infusing into lethally irradiated animals. The percentage of transplanted CFCs that homed into the BM within 16 h was plotted (*n*=4, *N*=12, *t* test: NS *P*>0.05). (**d**) Expression of Itgav (CD51) and Itgab3 (CD61) on SLAM KLS cells assessed by flowcytometry (*n*=6). (**e**) KLS cells incubated with SCF+TPO without (ST) or with (STP) Postn for 3 h were allowed to adhere on cyclo-RGDfK coated plates. The percentage of cells that adhered to the plates after 3 h was plotted for each condition (*n*=4, *t* test: *P=0.018). (**f**,**g**) BM derived KLS cells were cultured in serum-free medium containing SCF and TPO for 2 days without (ST) or with (STP) Postn alone, or in combination with neutralizing antibodies against Itgav (STP+αItgav) or Itgb3 (STP+αItgb3) for 2–5 days. (*n*=4, *t* test: **P<0.01; scale bar, 50 μm). (**f**) Bright field image showing cell expansion after 2 days. The proliferating cells appear as a cluster of cells in the middle of the round-bottom 96-well plates. (**g**) After 5 days of culture, cells were harvested and fold expansion was compared. (*n*=independent experiments, *N*=number of mice. Error bars indicate mean ±s.e.m.).

As improved homing potential could cause enhanced long-term engraftment of HSCs cultured with Postn, we first examined whether there was any change in adhesion and migration potential of Postn cultured cells *in vitro* (Fig. 4a,b). We found no differences in the adhesion of KLS progeny cultured with or without Postn to the BM stromal cell line ST2 (Fig. 4a). We also could not detect any change in their migration towards SDF-1α (Fig. 4b). To further assess BM homing of the Postn cultured cells, lethally irradiated mice were transplanted with the progeny of KLS cells cultured with or without Postn and the proportion of total colony-forming cells (CFCs) transplanted that homed in the BM within 16 h was quantified (Fig. 4c). We found no effect of Postn on the homing potential of KLS progeny.

We next determined if the Itgav (CD51) and Itgb3 (CD61) subunits, which make a heterodimer known to be a binding partner of Postn, were expressed on SLAM KLS cells (Fig. 4d). While more than 70% of SLAM KLS cells expressed Itgav, only 25% expressed Itgb3. Itgavb3 binds specifically to the cyclic Arg-Gly-Asp-D-Phe-Lys (cyclo-RGDfK) peptide^34^. To determine if Postn binds to Itgavb3, we examined the adhesion of KLS cells, incubated for 3 h with or without Postn, to cyclo-RGDfK coated ultra-low attachment plates (Fig. 4e). Cyclo-RADfK was used as negative control for each experiment. We found decreased attachment of KLS progeny cultured with Postn to cyclo-RGDfK coated plates, compared with control. We then examined if neutralizing Itgav or Itgb3 antibodies could block Postn-induced inhibition of proliferation of KLS cells (Fig. 4f,g). We found that Postn-induced decrease in expansion of HSPCs was inhibited by neutralization of Itgav but not Itgb3 (Fig. 4g). These results demonstrate that Postn binds to Itgav and that signalling via Itgav may be responsible for the inhibition of HSC proliferation caused by Postn.

### Decreased HSCs and myeloid bias in *Vav-Itgav* ^*−/−*^ mice

**Figure 5:**
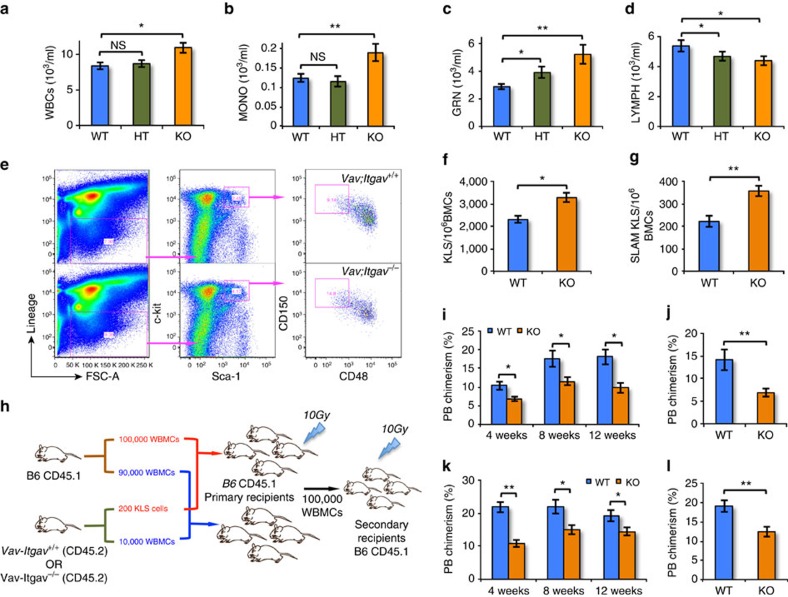
*Itgav*^*fl/fl*^*;Vav-icre* mice show decreased proportion of BM HSCs. (**a**–**d**) Counts for various blood cell types in *Vav-iCre*^*+*^*; Itgav*^*fl/fl*^ (KO), *Vav-iCre*^*+*^*; Itgav*^*fl/+*^ (HT) and *Vav-iCre*^*+*^*; Itgav*^*+/+*^ (WT) mice. WBC (**a**), monocyte (**b**), granulocyte (**c**) lymphocyte (**d**) counts in WT, HT and KO mice (*N*=10, *t* test: **P<0.01, *P<0.05). (**e**–**g**) Phenotypic analysis of BM cells by flowcytometry (**e**), to compare the numbers of KLS (**f**) and SLAM KLS (**g**) cells (*N*=12, *t* test: **P<0.01, *P<0.05). (**h**) Schematic representation of the competitive repopulation assay. 200 KLS cells or 10,000 WBMCs from *Vav;Itgav*^*−/−*^ mice (CD45.2) along with 100,000 or 90,000 whole BM competitor cells (CD45.1), respectively, were transplanted into sub-lethally irradiated CD45.1 WT CD45.1 mice. PB chimerism was followed for 12 weeks, after which secondary transplantations were performed. (**i**,**j**) PB chimerism in primary and secondary recipients transplanted with 10,000 total BM cells from *Vav-iCre*^*+*^*; Itgav*^*fl/fl*^ (KO) and *Vav-iCre*^*+*^*; Itgav*^*+/+*^ (WT) mice, together with 90,000 competitor cells (*n*=3, *N*=15, *t* test: **P<0.01, *P<0.05). (**k**,**l**) PB chimerism in primary and secondary recipients transplanted with 200 KLS cells from the BM of *Vav-iCre*^*+*^*; Itgav*^*fl/fl*^ (KO) and *Vav-iCre*^*+*^*; Itgav*^*+/+*^ (WT) mice, together with 100,000 competitor cells (*n*=3, *N*=12, *t* test: **P<0.01, *P<0.05). (*n*=independent experiments, *N*=number of mice. Error bars indicate mean ±s.e.m.).

As neutralizing antibodies against Itgav blocked the inhibitory effect of Postn on HSC proliferation *in vitro*, we wished to determine if the haematopoietic phenotype found in *Postn*^*−/−*^ mice would be phenocopied in mice with Itgav deletion. Universal deletion of Itgav causes perinatal lethality^35^. Therefore, we conditionally deleted *Itgav* in the haematopoietic system by crossing *Itgav*^*fl/fl*^ mice^36^ with *Vav-iCre* mice^37^ (Supplementary Fig. 4A,B). Litters were born in normal mendelian ratios and appeared healthy. We confirmed the lack of *Itgav* expression in BM cells by qRT-PCR (Supplementary Fig. 4C). Using flowcytometry, we confirmed that BM KLS cells did not express Itgav protein (Supplementary Fig. 4D). We next assessed PB counts in *Vav-iCre*^*+*^*;Itgav*^*fl/fl*^ mice (*Vav-Itgav*^*−/−*^; KO) in comparison with *Vav-iCre*^*+*^*;Itgav*^*fl/+*^ (*Vav-Itgav*^*+/−*^; HT) and *Vav-iCre*^*+*^*;Itgav*^*+/+*^ (*Vav-Itgav*^*+/+*^; WT) mice. In young adults (8 weeks old), we observed increased numbers of total WBCs (Fig. 5a) in *Vav-Itgav*^*−/−*^ mice with specifically elevated monocyte (Fig. 5b) and granulocyte (Fig. 5c) counts compared with *Vav-Itgav*^*+/+*^. Like in 16-week-old *Postn*^*−/−*^ mice, we observed a decreased lymphocyte count in 8-week-old *Vav-Itgav*^*−/−*^ as well as *Vav-Itgav*^*+/−*^ mice when compared with *Vav-Itgav*^*+/+*^ mice (Fig. 5d). We observed no change in RBC count (Supplementary Fig. 4E), haematocrit values (Supplementary Fig. 4F), haemoglobin levels (Supplementary Fig. 4G) and platelet counts (Supplementary Fig. 4H). Next, we compared the number of KLS and SLAM KLS cells in BM of 8-week-old KO and WT mice (Fig. 5e). We did not observe any change in total BM cellularity (Supplementary Fig. 4I); however, we observed an increase in the number of KLS (Fig. 5f) as well as SLAM KLS (Fig. 5g) cells. We performed competitive repopulation assays to assess the functional properties of *Itgav*^*−/−*^ HSCs (Fig. 5h). First, 10,000 *Vav-Itgav*^*−/−*^ or *Vav-Itgav*^*+/+*^ WBMCs along with 90,000 supporting CD45.1 WBMCs were transplanted into lethally irradiated CD45.1 recipients (Fig. 5i,j). As was also seen in *Postn*^−/−^ WBMC transplantations, we observed lower donor chimerism in primary (Fig. 5i) as well as secondary recipients (Fig. 5j) despite the increased number of SLAM KLS cells in the BM of *Vav-Itgav*^*−/−*^ mice. As increased granulocyte and decreased lymphocyte counts in PB was already very obvious at 8 weeks in *Vav-Itgav*^*−/−*^ mice, suggesting earlier HSC exhaustion in *Vav-Itgav*^*−/−*^ than in *Postn*^*−/−*^ mice, we tested whether purified HSCs from these mice had decreased repopulation potential (Fig. 5k,l). Competitive repopulation with 200 KLS cells from *Vav-Itgav*^*−/−*^ mice along with 100,000 CD45.1 WBMCs cells into lethally irradiated CD45.1 recipient mice resulted in a lower donor chimerism in primary (Fig. 5k) as well as secondary recipients (Fig. 5l). Transplantation of WBMCs as well as sorted HSCs from *Vav-Itgav*^*−/−*^ mice led to higher myeloid lineage reconstitution with reduced B- and T-cell lineage reconstitution (Supplementary Fig. 4J,K), consistent with the observations on PB cell counts from these mice. As haematopoietic system specific deletion of Itgav and systemic deletion of Postn has a very similar effect on haematopoiesis, this strongly supports our hypothesis that Postn-Itgav interaction is important for HSC maintenance.

### Postn-unresponsive *Vav-Itgav* ^*−/−*^ HSCs proliferate faster

**Figure 6:**
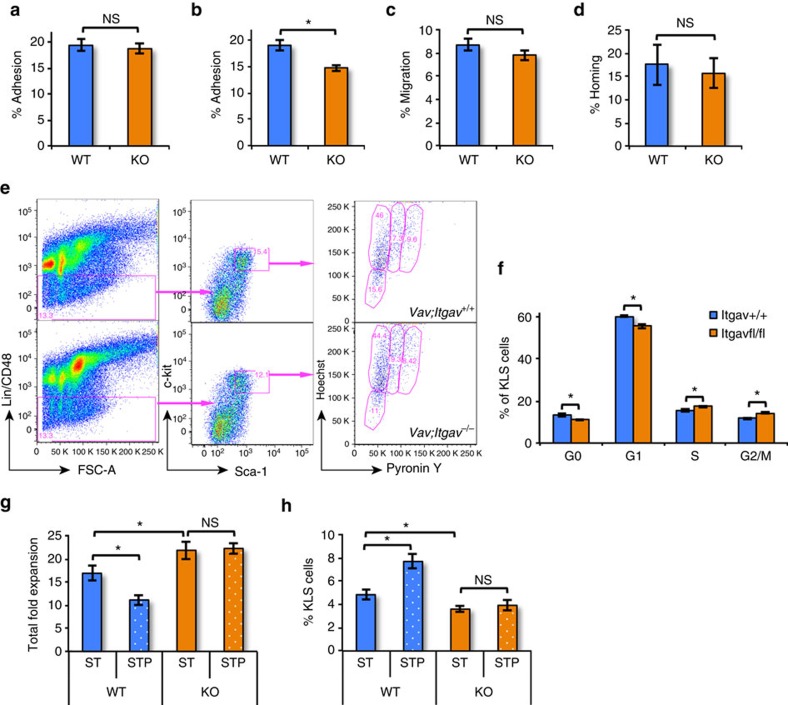
*Itgav* deficient HSPCs proliferate faster without any change in homing potential. (**a**) Fraction of lineage depleted *Vav;Itgav*^*+/+*^ (WT) or *Vav;Itgav*^*−/−*^ (KO) BM cells that adhered to ST2 cells within 3 h of incubation (*n*=4, *t* test: NS *P*>0.05). (**b**) Fraction of lineage depleted *Vav;Itgav*^*+/+*^ (WT) or *Vav;Itgav*^*−/−*^ (KO) BM cells that adhered to cyclo-RGDfK coated plates within 3 h of incubation (*n*=4, *t* test: *P=0.02). (**c**) Proportion of lineage depleted *Vav;Itgav*^*+/+*^ (WT) or *Vav;Itgav*^*−/−*^ (KO) BM cells that migrated towards SDF-1α in trans-well migration assays (*n*=4, *t* test: NS *P*>0.05). (**d**) Lineage depleted BM cells from *Vav;Itgav*^*+/+*^ (WT) or *Vav;Itgav*^*−/−*^ (KO) mice, were infused in irradiated animals. The percentage of transplanted CFCs that homed into the BM within 16 h was plotted (*n*=3, *N*=12, *t* test: NS *P*>0.05). (**e**) Cell cycle analysis of BM derived KLS cells from *Vav;Itgav*^*+/+*^ (WT) and *Vav;Itgav*^*−/−*^ (KO) mice by Hoechst 33342 staining in combination with Pyronin Y (*n*=6). (**f**) Proportion of KLS cells in WT and KO mice in different cell cycle stages (*n*=6, *t* test: *P<0.05). (**g**,**h**) KLS cells sorted from *Vav;Itgav*^*+/+*^ (WT) or *Vav;Itgav*^*−/−*^ (KO) mice BM were cultured in the presence of SCF and TPO with or without Postn in serum-free medium. Total fold expansion (**g**) and the proportion of KLS cells in expanded cells (**h**) were compared (*n*=6, *t* test: *P<0.05, NS *P*>0.05). (*n*=independent experiments, *N*=number of mice. Error bars indicate mean ±s.e.m.).

As loss of Itgav from HSC might lead to decreased homing of HSC and hence decreased repopulation, we performed *in vitro* adhesion (Fig. 6a,b) and migration assays (Fig. 6c), as well as *in vivo* homing assays (Fig. 6d). While, we did not observe any difference in their attachment to ST2 cells (Fig. 6a), we observed a modest decrease in adhesion of *Vav-Itgav*^*−/−*^ lin^−^ BM cells to RGDfK coated plates (Fig. 6b). Their migration towards SDF-1α was not affected compared with *Vav-Itgav*^*+/+*^ controls (Fig. 6c). *In vivo* assays did not show any difference in homing capacity of transplanted *Vav-Itgav*^*+/+*^ or *Vav-Itgav*^*−/−*^ haematopoietic progenitors (Fig. 6d).

A second explanation for the decreased repopulation of *Vav-Itgav*^*−/−*^ HSC might be loss of stemness due to enhanced proliferation caused by loss of the inhibitory effect of Postn in the BM niche. To test this hypothesis, we first performed Ho/Py staining to quantify the proportion of KLS cells in different stages of the cell cycle (Fig. 6e,f). This analysis revealed an increase in the number of KLS cells in the S as well as the G_2_/M phase, while the proportion of cells in the G_0_ and G_1_ phase was decreased (Fig. 6f). The anti-Itgav antibody blocking studies already suggested the importance of Itgav in mediating action of Postn on HSCs (Fig. 4f,g). This was further substantiated by studies wherein KLS cells from *Vav-Itgav*^*+/+*^ and *Vav-Itgav*^*−/−*^ mice were cultured with or without Postn (Fig. 6g,h). Consistent with the antibody blocking studies (Fig. 4f,g), *Vav-Itgav*^*+/+*^ KLS cells expanded less in the presence of Postn (Fig. 6g) and the proportion of KLS cells following 5 days of culture was higher in the presence of Postn (Fig. 6h). However, deletion of the Itgav receptor from the KLS cells prevented the inhibitory effect of Postn on cell proliferation (Fig. 6g), which was associated with a decreased frequency of KLS cells (Fig. 6h). Thus, loss of Itgav does not affect HSPC homing, but causes non-responsiveness to Postn, leading to excessive proliferation and differentiation of HSPC *in vivo* and *in vitro*.

### Postn induces p27kip1 expression by PI3K and Akt inhibition

**Figure 7:**
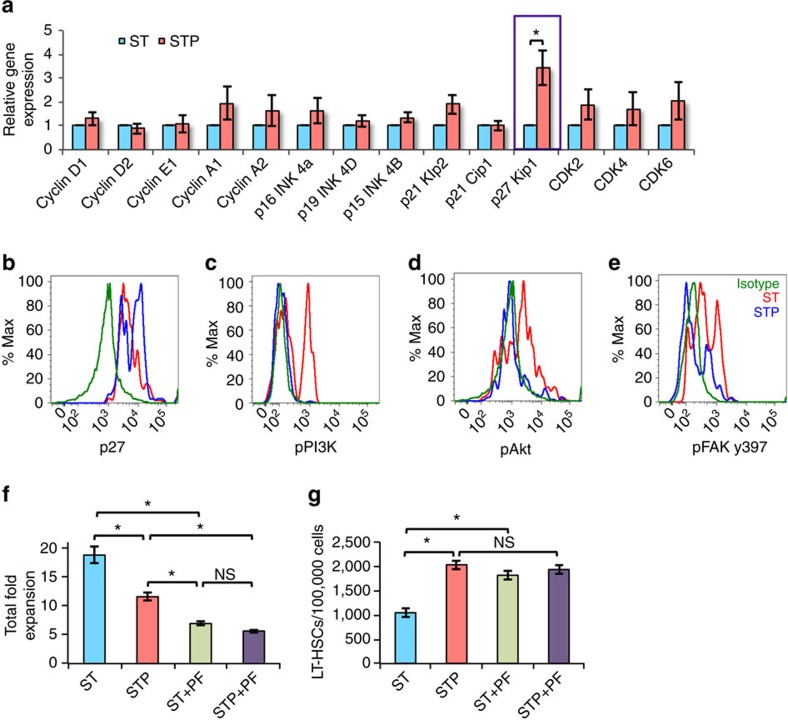
PI3K/Akt inhibition leading to p27kip1 expression inhibits HSC proliferation *in vitro*. (**a**) BM derived KLS cells cultured in serum-free medium with SCF+TPO without (ST) or with (STP) Postn were examined for expression of cell cycle regulatory genes by qRT-PCR (*n*=6, *t* test: *P=0.03). (**b**–**e**) Lineage depleted BM cells cultured for 2 days in serum-free medium SCF+TPO without (ST) or with (STP) Postn, were stained for cell surface markers to identify HSCs along with intracellular staining for p27kip1 (**b**) and phosphorylated forms of PI3K (**c**), Akt (**d**) and FAK y397 (**e**) (*n*=4). (**f**,**g**) BM derived KLS cells cultured in serum-free medium containing SCF and TPO for 2 days without (ST) or with (STP) Postn alone, or in combination with the FAK inhibitor PF-573228. After 5 days, cells were harvested and total cell expansion was measured (**f**). Harvested cells were stained for markers to identify LT-HSCs and the proportion of LT-HSCs in different culture conditions was examined (*n*=6, *t* test: *P<0.05, NS *P*>0.05) (**g**). (*n*=independent experiments, *N*=number of mice. Error bars indicate mean ±s.e.m.) (*n*=6, *t* test: *P<0.05, NS *P*>0.05).

**Figure 8:**
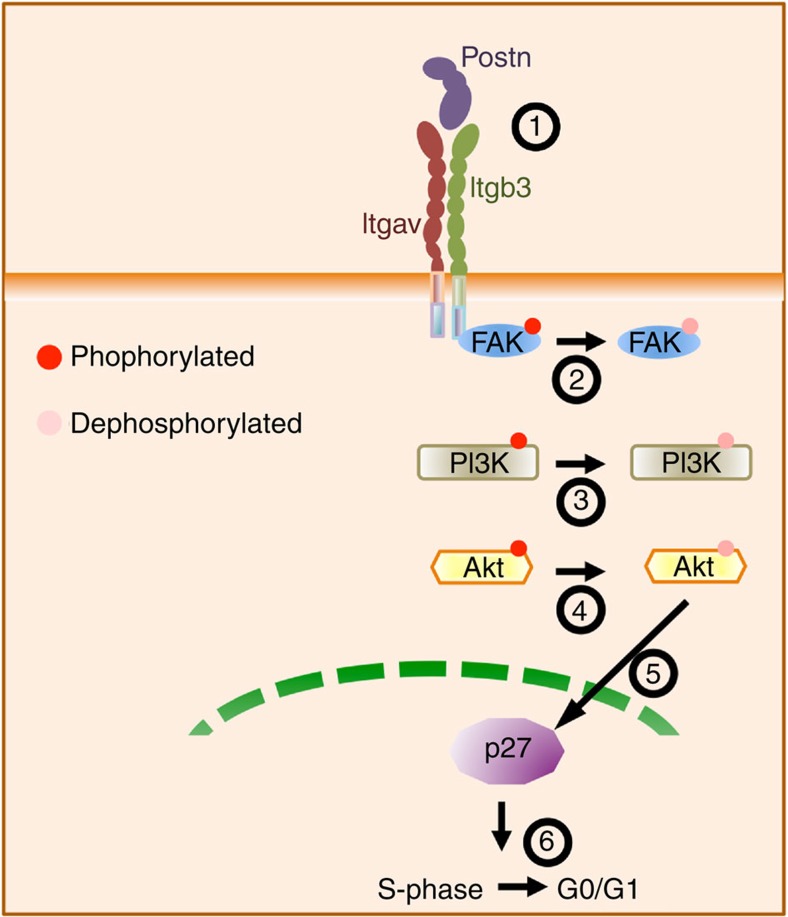
Schematic representation of the signalling events involved. Postn binding to its receptor Itgavb3 leads to dephosphorylation of FAK inhibiting PI3K/Akt pathways that regulates the expression and activity of p27Kip1. This pathway maintains the HSCs in quiescent state.

We then went on to address the molecular mechanism by which Postn binding to Itgav on HSCs inhibits their proliferation. We first tested the expression of various cell cycle regulators in the KLS cells cultured with or without Postn using qRT-PCR (Fig. 7a). Amongst all the genes tested (*CyclinD1*, *CyclinD2*, *CyclinE1*, *CyclinA1*, *CyclinA2*, *p16Ink4c*, *p19Ink4d*, *p15Ink4b*, *p57Kip2*, *Cdk2*, *Cdk4*, *Cdk6*, *p21Cip1*, *p27Kip1*) we found the expression of *p27Kip1* increased. We then performed experiments to confirm these results at the protein level. Lineage depleted BM cells were cultured with or without Postn and harvested after 2 days. We examined if Postn affected the expression of p27Kip1 protein in HSCs (Fig. 7b). Flowcytometry analysis for p27Kip1 expression demonstrated that Postn treatment led to elevated levels of p27kip1 in SLAM KLS cells (Fig. 7b). In a variety of cell types, the PI3K/Akt pathway has been shown to regulate cell proliferation by regulating the expression and activity of p27Kip1 (refs 38, 39). Therefore, we examined the phosphorylation status of PI3K (Fig. 7c) and Akt (Fig. 7d) in the SLAM KLS fraction of lin^−^ cells cultured with or without Postn using phospho-specific antibodies. We observed a decrease in phosphorylation of both PI3K and Akt, indicating that Postn inhibited the PI3K/Akt pathway leading to increased expression of p27Kip1 and decreased HSC proliferation. FAKs play important roles in transmitting outside-in integrin signals and act as phosphorylation-regulated signalling scaffolds^40^. FAKs have been shown to regulate several cellular functions such as proliferation, apoptosis and migration via the PI3K/Akt pathway^41^. We therefore examined the activation status of FAK in SLAM KLS cells, following culture of lin^−^ BM cells with Postn (Fig. 7e, Supplementary Fig. 5A) using phospho-specific antibodies to detect phosphorylation at different sites in FAK protein. While we did not observe any change in phosphorylation at tyrosine 576 or 577 (Supplementary Fig. 5A, left), there was decreased phosphorylation at tyrosine 397 (Fig. 7e). As some studies have demonstrated that outside-in integrin signalling results in activation of Src, which in turn can activate PI3K/Akt pathway, we also analysed if culture of HSPCs with Postn affected Src phosphorylation. We observed an increase in the phosphorylation status at the activating site (y416; Supplementary Fig. 5B) along with decreased phosphorylation at the de-activating site (y527, Supplementary Fig. 5C). As phosphorylation of both PI3K and Akt was decreased in Postn treated cells, these results indicated the involvement of FAK mediated integrin signalling rather than Src phosphorylation for cell cycle regulation in HSCs by Postn. Consistent with this hypothesis, we observed decreased overall expansion (Fig. 7f) with concomitant increase in the proportion of LT-HSCs (Fig. 7g) when KLS cells were cultured in the presence of a FAK inhibitor, PF-573228, even without addition of Postn. Collectively, these data suggests that binding of Postn to Itgav results in inhibition of the FAK/PI3K/Akt pathway, causing increased expression of p27kip1 and decreased HSC proliferation (Fig. 8).

### DNA damage accumulation in young *Postn* ^−/−^ HSCs

**Figure 9:**
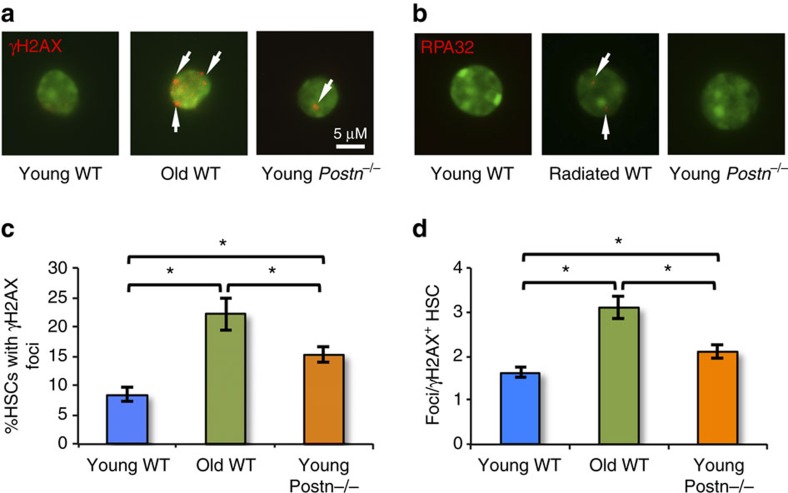
HSCs in young *Postn*^*−/−*^ mice exhibit DNA damage accumulation. (**a**). Representative primitive HSCs (SLAM KLS cells) isolated by FACS and stained with anti-γH2AX antibodies (pseudo-color red) and Hoechst 33342 (pseudo-color green). White arrows indicate foci. (*n*=4). (**b**). Representative example of primitive HSCs (SLAM KLS cells) isolated by FACS and stained with anti-RPA antibodies (pseudo-color red) and Hoechst 33342 (pseudo-color green). White arrows indicate foci. (*n*=4). (**c**). Percentage of HSCs with γH2AX-marks from young *Postn*^*−/−*^ mice (right), young WT (left), and old WT (middle) mice. (*n*=4, *t* test: *P<0.05). (**d**). Average number of γH2AX-positive foci in primitive HSCs from young *Postn*^*−/−*^ mice (right), young WT (left) and old WT (middle) mice. (*n*=4, *t* test: *P<0.05). (*n*=independent experiments, Error bars indicate mean ±s.e.m.).

Accumulation of DNA damage has been associated with replicative stress, which in turn has been implicated in several age associated haematopoietic disorders^42^. In aged mice, impaired DNA damage responses have been observed in HSCs^43^,^44^. In addition, HSCs with a defect in the DNA damage response pathways, such as in *Atm* deficient mice, function poorly^45^. Our results showed that interrupting the Postn-Itgav interaction in *Postn*^*−/−*^ as well as *Vav-Itgav*^*−/−*^ mice, led to poor functioning of HSCs concomitant with faster rate of proliferation and functional decline. Therefore, we tested if HSCs from young (16 week old) *Postn*^*−/−*^ mice showed DNA damage accumulation. SLAM KLS cells from young (16 week old) and old (18 month old) WT mice as well as young (16 week old) *Postn*^*−/−*^ mice were sorted and immuno-stained using γH2AX antibodies to identify DNA damage marks (Fig. 9a). First, the proportion of cells with γH2AX foci (at least one) was quantified. Analysis clearly revealed increased proportion of HSCs from young *Postn*^*−/−*^ mice with γH2AX foci compared with young WT mice (Fig. 9b). As expected, higher proportion of SLAM KLS cells from old WT mice contained γH2AX foci. We also quantified the extent of DNA damage accumulation in HSCs from each source by enumerating the number of γH2AX foci in individual SLAM KLS cells, isolated from young as well as old WT mice, and young *Postn*^*−/−*^ mice (Fig. 9c). We observed increased level of γH2AX foci accumulated in individual HSCs from young *Postn*^*−/−*^ mice compared with young WT mice. Again, the highest level of DNA damage accumulation was observed in HSCs from old mice. To exclude the possibility that appearance of γH2AX^+^ foci could be caused by replication arrest^46^, we also performed replication protein A (RPA) staining on primitive HSCs isolated from WT or *Postn*^*−/−*^ mice (Fig. 9d). Unlike in ultraviolet irradiated control samples, we did not observe RPA staining in either WT or *Postn*^*−/−*^ mouse derived HSCs. Thus, greater DNA damage accumulation in HSCs from young *Postn*^*−/−*^ mice compared with WT mice suggests that HSC may undergo functional decline as a result of the replicative stress in these cells.

## Discussion

We present data demonstrating the importance of Postn-Itgavb3 interaction in the regulation of murine HSC function. The BM of *Postn*^*−/−*^ mice contained increased numbers of SLAM KLS cells, which were more proliferative than the WT HSCs. Nevertheless, *Postn*^*−/−*^ mice developed progressive anaemia, myelomonocytosis and lymphopenia, and *Postn*^*−/−*^ BM cells repopulated the haematopoietic system following grafting poorly. We confirmed these results by haematopoietic tissue-specific deletion of Itgav, which acts a receptor for Postn. *Vav-Itgav*^*−/−*^ mice developed pronounced myelomonocytosis and lymphopenia at an earlier age than *Postn*^*−/−*^ mice. As was seen in *Postn*^*−/−*^ mice, *Vav-Itgav*^*−/−*^ BM contained elevated levels of SLAM KLS cells and hyperproliferative KLS cells. In addition, WBMCs and purified HSCs from *Vav-Itgav*^*−/−*^ mice poorly reconstituted the haematopoietic system following transplantation. Finally, we demonstrated that the loss of Postn-Itgavb3 interaction caused enhanced HSPC proliferation because of deactivation of the FAK/PI3K/AKT pathway responsible for regulating p27Kip1 expression, leading to increased proliferation and reduced stemness of HSCs in *Postn*^*−/−*^ as well as *Vav-Itgav*^*−/−*^ mice.

Proliferation and quiescence have been strongly linked to stemness in the haematopoietic system, as well as other stem cell models^47^,^48^. For instance, in *p21Cip1*^*−/−*^ mice, HSPC proliferation is increasingly reflected in increased numbers of phenotypically defined HSCs in the BM, with, however, loss of cells with long-term culture initiating capacity and long-term repopulating stem cells^32^. Similar changes have been observed in the haematopoietic system of mice wherein the growth factor independent 1 (*Gfi-1*)^49^ or mammalian target of rapamycin (*mTOR*)^50^, two upstream regulators of *p21Cip1*, were deleted from the haematopoietic system. In both of the models, increased proliferation of HSCs, with increased frequency of phenotypically defined HSCs linked with reduced repopulation activity was observed. The discrepancy between the increased number of phenotypic HSCs, identified as SLAM KLS cells, present in the BM of these mice, and their poor repopulation ability is also seen in aged animals^51^. These mice, like the mice deficient in *p21Cip, Gfi-1* or *mTOR* expression, also display a considerable bias towards myeloid lineage repopulation while poorer lymphoid chimerism was observed. Entry of quiescent HSCs into the cell cycle leads to loss of stemness, which leads to their faster exhaustion^52^,^53^. Recent studies using *Sirt1* deleted mice, demonstrated that extensive proliferation leads to pre-mature aging and loss of function in HSCs^54^.

We demonstrated that the deficiency of Postn leads to increased proliferation of HSPCs, and an increase in myelopoiesis and anaemia as early as 8 weeks after birth, which became more pronounced by 16 weeks. At 16 weeks of age, we also found a decreased lymphopoiesis, which combined with the increased myelopoiesis seen, is typical for a poorly functioning haematopoietic system and is also observed during aging^55^. In addition, purified primitive HSCs from 16-week-old *Postn*^*−/−*^ mice exhibited lower LT-repopulation potential and secondary recipients were poorly engrafted. This effect was also seen when the mice were transplanted with WBMCs from 8-week-old *Postn*^*−/−*^ mice, even though the proportion of phenotypic primitive HSCs was higher in WBMCs of *Postn*^*−/−*^ mice. These results suggest very strongly that the engraftment potential of the HSCs from *Postn*^*−/−*^ mice is poorer than from WT mice. Interestingly, we also observed a decrease in ST-repopulation in primary recipients when WBMCs from 8-week-old mice were transplanted showing that the engraftment efficiency of ST-HSCs was also affected in *Postn*^*−/−*^ mice. However, when primitive HSCs from 16-week-old mice were transplanted, no differences in the ST- repopulation were seen primarily because of lack of ST-HSCs in the transplanted population. In addition, faster proliferation of donor-derived HSC population in secondary recipients would compensate for ST-HSCs.

Postn is one of the ligands for Itgav-b3 expressed on HSCs. Because blocking Itgav but not Itgb3 with neutralizing antibodies inhibited the effect of Postn on HSCs *in vitro*, we tested whether the haematopoietic defects seen in *Postn*^*−/−*^ mice would be recapitulated in mice wherein Itgav was specifically deleted in haematopoietic cells. As seen in the *Postn*^*−/−*^ mice, accelerated loss of stemness was also observed in *Vav-Itgav*^*−/−*^ mice. In fact the degree of myeloid skewing was more pronounced in *Vav-Itgav*^*−/−*^ mice, compared with *Postn*^*−/−*^ mice. In both *Postn*^*−/−*^ as well as *Vav-Itgav*^*−/−*^ mice, an increase in SLAM KLS cells was found in the BM as early as 8 weeks after birth. However, when whole BM from either 8-week-old *Postn*^*−/−*^ or *Vav-Itgav*^*−/−*^ mice (young adults) was grafted, decreased long-term repopulation was observed.

There is an extensive body of evidence that engagement of integrins, such as integrin-α4β1, affect HSC function^56^. Conditional deletion of integrin-α4 (Itga4) in the haematopoietic system resulted in increased circulation of progenitors due to severe loss of HSC maintenance within the BM^57^. In addition, HSCs that do not express Itga4 repopulate the haematopoietic system very poorly due to loss of homing potential. Here, we demonstrate that loss of Itgav from HSC also caused poorer HSC engraftment. But, in contrast to the studies on Itga4, loss of engraftment of *Vav-Itgav*^*−/−*^ HSC was not caused by defects in HSC homing. Recent studies demonstrated a role for Itgb3 in HSC maintenance^10^,^11^. These studies demonstrated that inside-out signalling from the TPO receptor activates Itgb3 integrin, which in response to extracellular signals causes an outside-in increased TPO-dependent enhanced haematopoietic reconstitution of HSCs. However, if the loss of Itgb3 expression or its outside-in signalling activity, affects HSC proliferation has not yet been reported.

Our *ex vivo* studies demonstrated that inhibition of HSC proliferation by Postn, results from binding of Postn to Itgav. Culture of KLS cells in the presence of Postn decreased total cell expansion with, however, preservation of a greater fraction of primitive HSCs. Importantly, Postn did not affect the proliferation of Itgav deficient HSCs, confirming the specificity of the interaction. In addition, Itgav deficient KLS cells proliferated faster than control cells *ex vivo* as they did *in vivo* in *Postn*^*−/−*^ as well as in *Vav-Itgav*^*−/−*^ mice. Likewise, HSC from *Postn*^*−/−*^ as well as in *Vav-Itgav*^*−/−*^ mice also proliferated more *in vivo* compared with WT HSCs. We further demonstrated that the faster cycling of *Postn*^*−/−*^ HSCs led to faster recovery of the haematopoietic system from radiation injury. However, consistent with the notion that excessive proliferation of HSCs causes exhaustion of the HSC pool, we found depletion of the HSCs in the BM of irradiated animals 8 weeks following the radiation injury.

Accumulation of DNA damage is the hallmark feature of replicative stress in stem cells^58^. It was recently shown that induction of exit from homeostatic state of dormancy in HSCs directly results in DNA damage accumulation^59^. As both *Postn*^*−/−*^ as well as *Vav-Itgav*^*−/−*^ mice contained lower proportions of quiescent HSCs and the haematopoietic progenitors proliferated faster, we tested if this was associated with DNA damage accumulation. Our observation that greater proportion of HSCs from young *Postn*^*−/−*^ mice had accumulated DNA damage, demonstrated by an increase in the frequency of γH2AX^+^ foci, but not RPA32 foci, therefore confirms our hypothesis that the loss of Postn-Itgav interactions caused poor HSC function.

Postn binds to heterodimers of Itgav and multiple β-chains in a variety of cell types^16^. As discussed above, the effect of Postn on the proliferation of KLS cells *in vitro* could be blocked only with antibodies against the Itgav chain, not the Itgb3 chain, strongly suggesting that Itgav is responsible for binding of Postn to the heterodimer, while outside-in signalling must occur via the Itgb3 chain. In vascular smooth muscle cells, binding of Postn to Itgavb3 induced phosphorylation of FAK (ref. 16). In contrast, we found reduced phosphorylation of FAK at Tyr397 in SLAM KLS cells in response to Postn *in vitro*. We demonstrated that inactivation of FAK was associated with inactivation of the PI3K/Akt pathway, which is known to regulate p27Kip1 expression and activity^60^,^61^. Inhibition of FAK phosphorylation by the small molecule inhibitor, PF-573228, phenocopied the effect of Postn on the *in vitro* expansion of KLS cells. Noteworthy, among all the cell cycle regulators tested, the expression of p27kip1 was found to be enhanced in HSPCs treated with Postn. Loss of p27Kip1 leads to increased proliferation of haematopoietic progenitors^28^, even if the number of long-term repopulating HSC is not affected. Deletion of *p57Kip2* in *p27kip1*^*−/−*^ HSCs, however, resulted in a decrease in their long-term self-renewal of long-term culture initiating cells^62^. Our results strongly suggest the involvement of p27Kip1 in Postn-Itgav-mediated regulation of HSC proliferation, but do not rule out the involvement of other factors in this process. Outside-in signalling from integrins can also involve activation of Src, which then activates the PI3K/Akt pathway^63^. We did indeed observe Src activation, but this in the presence of dephosphorylated PI3K and Akt, indicating that Src activation by Postn-Itgav binding is not responsible for the decreased proliferation observed.

In conclusion, these studies establish Postn-Itgav interaction as an important regulator of HSC proliferation. We provide evidence that outside-in signalling from Itgavb3 in response to Postn causes inactivation of FAK-induced PI3K/Akt signalling, leading to higher expression of the cell cycle regulator p27kip1, which has been shown to induce HSC quiescence^28^. Either the systemic loss of Postn or haematopoietic specific deletion of Itgav causes excessive proliferation under physiological conditions *in vivo*, or following injury, causing functional decline and exhaustion of the HSC pool.

## Methods

### Animals

Six- to eight-week-old FVB/NJ, C57BL/6J-CD45.2 (*Centre d'Elevage* R. *Janvier,* Le Genest-St Isle*, France),* B6.SJL-PTPRCA-CD45.1 (Charles River Laboratories, Raleigh, NC), *Rag2*^*−/−*^*γC*^*−/−*^ (from Prof. Chantal Mathieu, Clinical and Experimental Endocrinology, UZ Leuven, Leuven, Belgium) and *Postn*^*−/−*^ mice^64^ were maintained in the animal facility at KU Leuven. *Itgav*^*flox/flox*^ mice^36^ were crossed to Vav-iCre mice (from Thomas Graf, Centre for Genomic Regulation, Barcelona) to obtain *Vav-iCre; Itgav*^*fl/fl*^ mice. Genotyping, as described before, was performed on genomic DNA from tail tips as well as from BM cells. *Vav-iCre*^*−*^*; Itgav*^*fl/fl*^ and *Vav-iCre*^*+*^*; Itgav*^*+/+*^ littermates were used as controls. During the experiments, mice were maintained in isolator cages, fed with autoclaved acidified water and irradiated food *ad libitum*. All experiments were approved by the Institutional Ethics Committee of KU Leuven.

### Haematopoietic stem cell sorting and culture

BM cells were flushed from femurs and tibiae of mice, pooled, washed twice with phosphate-buffered saline (PBS; Gibco Invitrogen Corp., Carlsbad, CA) containing 0.1% bovine serum albumin (Sigma, St Louis, MO) and filtered through a 40-μm nylon mesh. Lineage positive cells were depleted by magnetic-activated cell sorting (Miltenyi Biotech, Bergisch Gladbach, Germany). The Lineage depleted BM cells were stained with fluorescein isothiocyanate (FITC) conjugated anti-Sca-1, phycoerythrin (PE) conjugated anti-c-kit, allophycocyanin (APC) conjugated anti-Lineage antibody cocktail (0.5 μg ml^−1^ for each antibody; BD Pharmingen, San Diego, CA, USA). 7-amino actinomycin D (7-AAD; 0.1 μg per sample; Sigma, St Louis, MO) staining was performed to identify viable cells. KLS cells, which were 7-AAD negative, were sorted on a BD FACS Aria III (BD Biosciences, Mountain View, CA). The sorted cells were cultured in round-bottom 96-well plates (BD Biosciences, San Jose, MA) in 100 μl of Stemspan (Stem Cell Technologies) supplemented with 100 ng ml^−1^ mTPO and 50 ng ml^−1^ mSCF, with or without rmPostn (0.5–5 mg ml^−1^; R&D Systems, Minneapolis, MN). Cells were cultured for up to 5 days at 37 °C with 5% CO_2_.

### Competitive repopulation assays

Progeny of 200 CD45.1 KLS cells cultured with/without Postn were transplanted along with 100,000 competitor CD45.2 BM cells. For comparing *Vav-iCre*^*+*^*; Itgav*^*fl/fl*^ with *Vav-iCre*^*+*^*; Itgav*^*+/+*^ repopulating cells, 200 freshly isolated KLS cells or 10,000 whole BMCs were transplanted in 8–12-week-old female mice along with 100,000 or 90,000 WBMCs, respectively. Whole BMCs (50,000) derived from *FVB/NJ* and *Postn*^*−/−*^ mice were transplanted in sub-lethally (3.5 Gy) irradiated *Rag2*^*−/−*^*γC*^*−/−*^ mice. For functional analysis of primitive HSCs CD150^+^CD48^−^KLS cells were fluorescence-activated cell sorting (FACS) sorted from *FVB/NJ* and *Postn*^*−/−*^ mice derived BM cells and 200 cells were transplanted in sub-lethally (3.5 Gy) irradiated *Rag2*^*−/−*^*γC*^*−/−*^ mice. PB multilineage chimerism analysis was performed every 4 weeks. After 12 weeks, primary recipients were killed; BM harvested and 100,000 (for *Vav-Itgav*^*−/−*^) or 1 × 10^6^ (for *Postn*^*−/−*^) cells grafted in lethally irradiated secondary recipients. After 3 months, multilineage chimerism in secondary recipients was evaluated. BM of the secondary recipients engrafted with sorted primitive HSCs derived from *FVB/NJ* and *Postn*^*−/−*^ mice was also used for analysis of frequency of donor-derived HSCs. In addition, cell cycle of donor-derived HSCs was performed by additional cell surface staining along with Ho/Py staining.

### Radiation recovery experiments

FVB/NJ and *Postn*^*−/−*^ mice were irradiated sub-lethally (5 Gy). The extent of haematopoietic injury as well as the recovery was monitored by PB analysis at 1-week intervals for 8 weeks on a scil Vet ABC animal blood counter (Horiba ABX, Montpellier, France). PB levels of RBCs, haematocrit, haemoglobin, total WBCs, monocytes, granulocytes, platelets and lymphocytes were measured.

### Flow cytometry

Chimerism and lineage analysis of BM derived cells was performed by flow cytometry. Lineage specific antibodies used were FITC conjugated Mac-1 and Gr-1 for myeloid cells, PECy7 conjugated B220 for B-cells, PE conjugated anti-CD4 and anti-CD8 for T-cells were used in addition to APC conjugated anti-CD45.1 and PerCPCy5.5 conjugated anti-CD45.2 antibodies. All antibodies were procured from BD Pharmingen at were used at 0.25 μg ml^−1^ concentration. Flow cytometric analysis for primitive HSCs and haematopoietic progenitors was performed using anti-mouse CD48 APC and CD150 PECy7 (0.25 μg ml^−1^; ebiosciences) along with KLS cells staining (as for sorting). Itgav and Itgb3 expression in the BM derived HSCs was analysed by using α-mItgav and α-mItgb3 along with primitive HSC markers. For intracellular staining, cells already labelled with antibodies against cell surface antigens were fixed with 4% paraformaldehyde, followed by permeabilization with 0.2% saponin. Antibodies against p27kip1 and phospho-specific antibodies against FAK, PI3K, Akt and Src were purchased from Cell signaling Technology Inc. (Beverly, MA, USA), and used at a 1 μg ml^−1^ concentration. Suitable isotype controls for each antibody were used in all experiments. The cells were analysed by flow cytometry using FACS Canto (BD Biosciences, San Jose, CA).

### Cell cycle analysis

BM derived cells were first stained for HSPC markers (Lineage, Sca-1, c-kit) followed by Hoechst 33342 (Ho) alone or in combination with Pyronin Y (PY) staining as described before^65^. For cell cycle analysis of donor-derived HSCs in secondary recipients, additional staining for CD45.1 was also performed. The cells stained for cell surface markers were incubated with Ho (10 μg ml^−1^) at 37 °C for 45 min. For Ho/Py staining, this was followed by incubation with 1 μg ml^−1^ PY and the cells were incubated for additional 30 min. Cell were acquired on FACS Aria III (BD Biosciences) and analysed using FlowJo software (TreeStar, Ashland, OR).

### BrdU incorporation assays

BrdU incorporation assays were performed as described before^66^. Briefly, 1 mg of BrdU per animal was injected intra-peritoneally for 3 (for KLS cells) or 7 (for LT-HSCs) days before analysis. After 3 or 7 days, mice were killed and BM derived cells were analysed for BrdU incorporation with in KLS cell population identified by cell surface staining. Cells stained in the same manner but isolated from an uninjected mouse were used in all cases as negative controls for BrdU staining. The proportion of KLS cells with BrdU uptake was enumerated.

### *In vitro* adhesion assays

For cell adhesion assays, the cells were first labelled with the PKH-26 membrane dye^67^ according to the manufacturer's instructions (Sigma, St Louis, MO, USA). Freshly sorted or cultured KLS cells were harvested and washed with PBS to remove any protein content. The cells were re-suspended at 1 × 10^7^ per ml of Diluent C. The cell suspension was mixed with an equal volume of 4 mM PKH-26 dye (in Diluent C) for 5 min at room temperature. An equal volume of foetal bovine serum (FBS) was added to stop the reaction and the cells were washed with medium containing 10% FBS. 5 × 10^4^ ST2 cells were plated per well in 24-well plates. 2 × 10^4^ PKH-26 labelled KLS progeny were added per well and incubated for 3 h at 37 °C and 5% CO_2_. Non-adherent cells were removed and adherent cells were harvested along with the feeder layer. Flowcytometry was used to quantify the labelled cells to compare cell attachment^68^. Results are represented as percentage of cells adhered.

### *In vitro* migration assays

*In vitro* trans-well migration assays were performed as described before with slight modifications^69^. 100,000 lineage depleted BM cells in 200 μl of chemotaxis buffer (RPMI 1640, 1% FBS; (Gibco BRL, Grand Island, NY), and antibiotics) were added to the upper chambers of a 6.5 mm, 3 μm pore size Transwell (Costar, Cambridge, MA) while the lower chambers containing 500 μl of 200 ng ml^−1^ SDF-1α. Chambers were incubated at 37 °C, 5% CO_2_ for 3 h. Cells migrating into the lower chamber were counted using FACS (FACS Canto, Becton Dickinson). Per cent migration was determined by calculating the per cent of input cells migrated into the lower chamber.

### *In vivo* homing assay

A protocol published earlier^30^ originally adapted from previously published reports^70^,^71^ was used to examine the homing potential of BM derived HSPCs. One day before transplantation, 6–8-week-old B6.SJL-PTPRCA-CD45.1 mice underwent total body lethal irradiation (10 Gy). 2 × 10^6^ BM cells from Vav-Itgav^+/+^ or Vav-Itgav^−/−^ mice were injected intravenously into the irradiated mice. Before transplantation, the frequency of CFCs in the transplanted cells were quantified. 16 h after injection, BM from transplanted mice was harvested and donor-derived HSPCs homed in the recipient BM were quantified by colony-forming unit-C assay. For each experiment, a set of 6 mice irradiated but not transplanted was used to quantify left over CFCs in the BM of the irradiated mice. Though negligible (10.64±4.54 per mouse), the values were taken into consideration for making calculation as described below. For CFC assays, 1 × 10^5^ cells were plated in 3 ml methylcellulose using MethoCult GF M3434 medium (Stem Cell Technologies). Each sample was cultured in triplicates at 37 °C with 5% CO_2_ in air. CFCs were scored after 9–12 days by light microscope. The proportion of total CFCs transplanted that homed in the total recipient BM represents homing.

### Quantitative RT-PCR

Total RNA was prepared using the RNA Isolation Kit (Qiagen, Hilden, Germany) according to the manufacturer's protocol. DNase treatment of RNA was performed using Turbo DNase kit (Ambion, Austin, TX, USA). The purity and the concentration of RNA were assessed using a ND-1000 spectrophotometer (NanoDrop Technologies, USA). 100 ng–1 μg of RNA from each sample was used to synthesize cDNA using Superscript III First-Strand Synthesis System (Invitrogen, Carlsbad, CA) according to the manufacturer's protocol. qRT-PCR was carried out using Taqman SYBR green universal mix PCR reaction buffer (Applied Biosystems, Foster City, CA). The PCR reactions were carried out in a mastercycler realplex (Eppendorf, Hamburg, Germany) using the following programme: 2 min at 50 °C, 1 min at 95 °C and 40 cycles of 30 s at 95 °C and 30 s at 60 °C. Amplified products from qRT-PCR performed on HSC niche components were also electrophoresed on agarose gel (1.5% in TAE buffer; Sigma Aldrich). The list of primers used is given in Supplementary Table 1.

### Immunostaining and fluorescence microscopy

FACS-sorted long-term HSCs (CD150^+^CD48^−^ KLS cells) were suspended in Stemspan medium. 200 cells per well were plated on fibronectin coated Teflon-printed 10-well glass slides (Matsunmai Glass Industry, Osaka, Japan) as described before^30^. The cells were fixed for 15 min in 4% paraformaldehyde, permeabilized in 0.1% Triton-X-100 (Sigma) and incubated for 1 h in blocking buffer (10% donkey serum/0.1% Triton-X-100/PBS). The cells were stained with anti-γH2AX or RPA34 antibodies conjugated with alexafluor-488 (2.5 μg ml^−1^) in addition to Hoechst 33342. Images were captured on a Zeiss AxioImager microscope (Zeiss, Jena, Germany) fitted with an AxioCam MRc5 digital camera. Images were captured using AxioVision AC software (Zeiss) and assembled in Adobe Photoshop.

### Statistical analysis

Data are shown as mean±s.e.m. Statistical analysis was performed using a two-tailed Student's *t* test. *P*-values <0.05 were considered statistically significant.

### Data availability

The authors declare that all data supporting the findings of this study are available within the article and its Supplementary Information Files or from the corresponding author upon reasonable request.

## Additional information

**How to cite this article:** Khurana, S. *et al*. Outside-in integrin signalling regulates hematopoietic stem cell function via Periostin-Itgav axis. *Nat. Commun.* **7,** 13500 doi: 10.1038/ncomms13500 (2016).

**Publisher's note**: Springer Nature remains neutral with regard to jurisdictional claims in published maps and institutional affiliations.

## Supplementary Material

Supplementary Information Supplementary Figures 1-5, Supplementary Table.
