# Supplementary material for: Outside-in integrin signalling regulates haematopoietic stem cell function via Periostin-Itgav axis
Source: Nat Commun. 2016 Dec 1;7:13500. doi: 10.1038/ncomms13500 (PMC5146274; doi:10.1038/ncomms13500)
Supplement: Supplementary Information — Supplementary Figures 1-5, Supplementary Table. [file ncomms13500-s1.pdf]

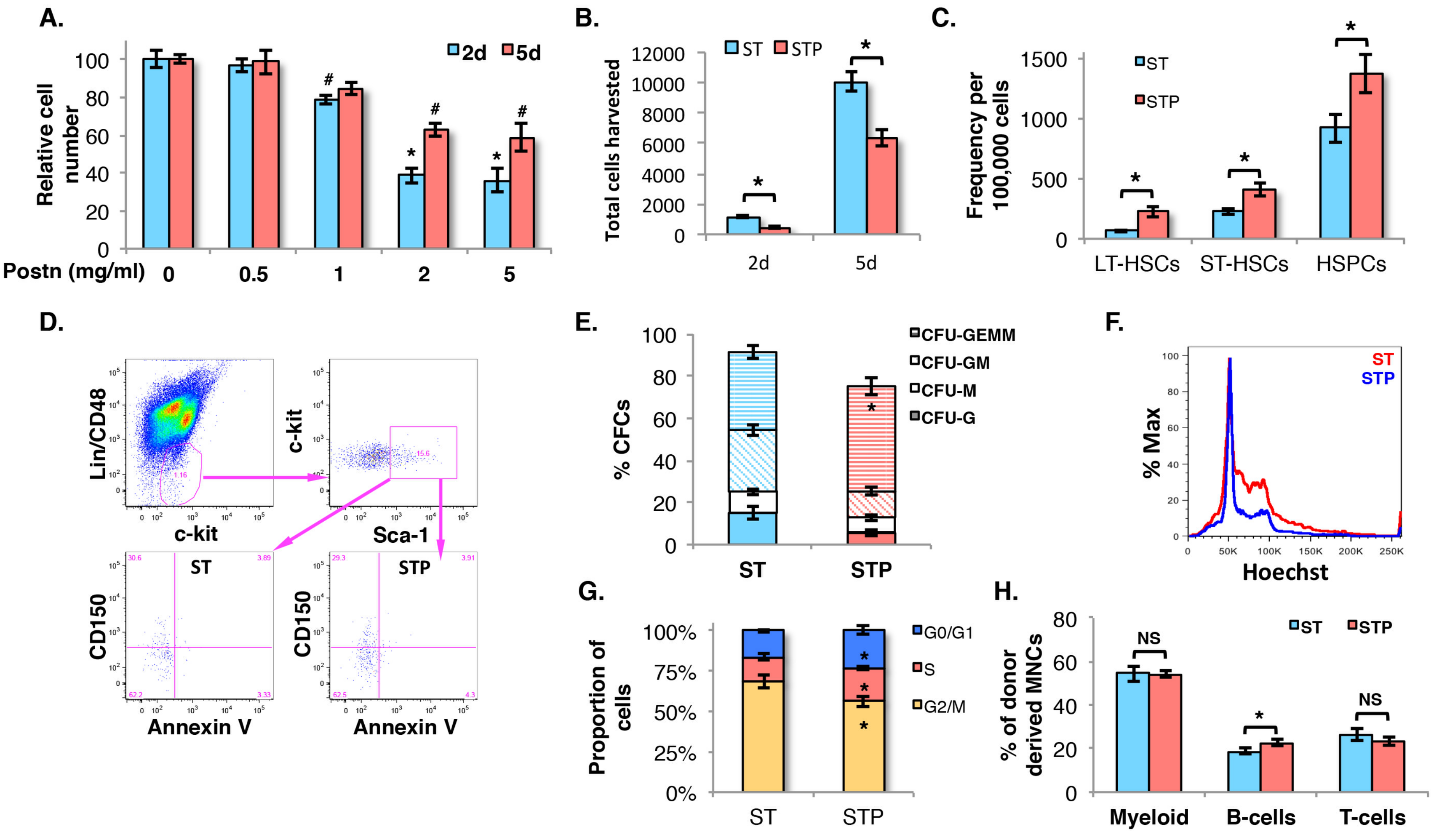

Supplementary Figure 1. Postn inhibits culture-induced proliferation of BM HSCs

(A) BM derived KLS cells were cultured in serum free medium in the presence of SCF and TPO with/without Postn (0-5mg/ml). Total cell number after 2-5 days of culture was compared using Nucleocounter NC100 (n=6, t test: * p<0.05).

(B) The total number of cells harvested after 2 and 5 days of culture of KLS cells. Cells were cultured in serum free medium in the presence of SCF and TPO with/without Postn (2mg/ml) (n=5, t test: * p<0.05).

(C) Cells harvested after 5 days of culture were analyzed for HSPC sub-populations by flowcytometry. Absolute frequencies of the sub-populations in the harvested cells are plotted (n=5, t test: * p<0.05)

(D) Lineage depleted BM cells cultured for 2 days in the presence of SCF and TPO in serum free medium with/without Postn, were stained for cell surface markers to compare the proportion SLAM KLS cells that co-expressed Annexin V.

(E) Methyl cellulose colony forming assays were performed using the cells harvested after 5 days of culture of KLS cells in the presence or absence of Postn. Frequency of CFCs was compared across different samples (n=5, t test: * p<0.001).

(F) Hoechst staining to compare proliferation of harvested KLS cell progeny. After 5 days of culture the cells were harvested and stained with Hoechst 33342. The proportion of day 5 KLS cell progeny in G_0 as quantified by Hoechst 33342/Pyronin Y staining (n=3).

(G) Cell cycle status of the KLS cells within the total harvested cell following culture. BM derived KLS cells were cultured in serum free medium in the presence of SCF and TPO with/without Postn (0-5mg/ml) for 5 days. After 5 days of culture the cells were harvested and stained with Hoechst 33342 in addition to the HSC markers (n=3).

(H) Multi-lineage engraftment analysis in peripheral blood in secondary recipients that received KLS cells cultured with/without Postn (n=12, t test: * p=0.03).

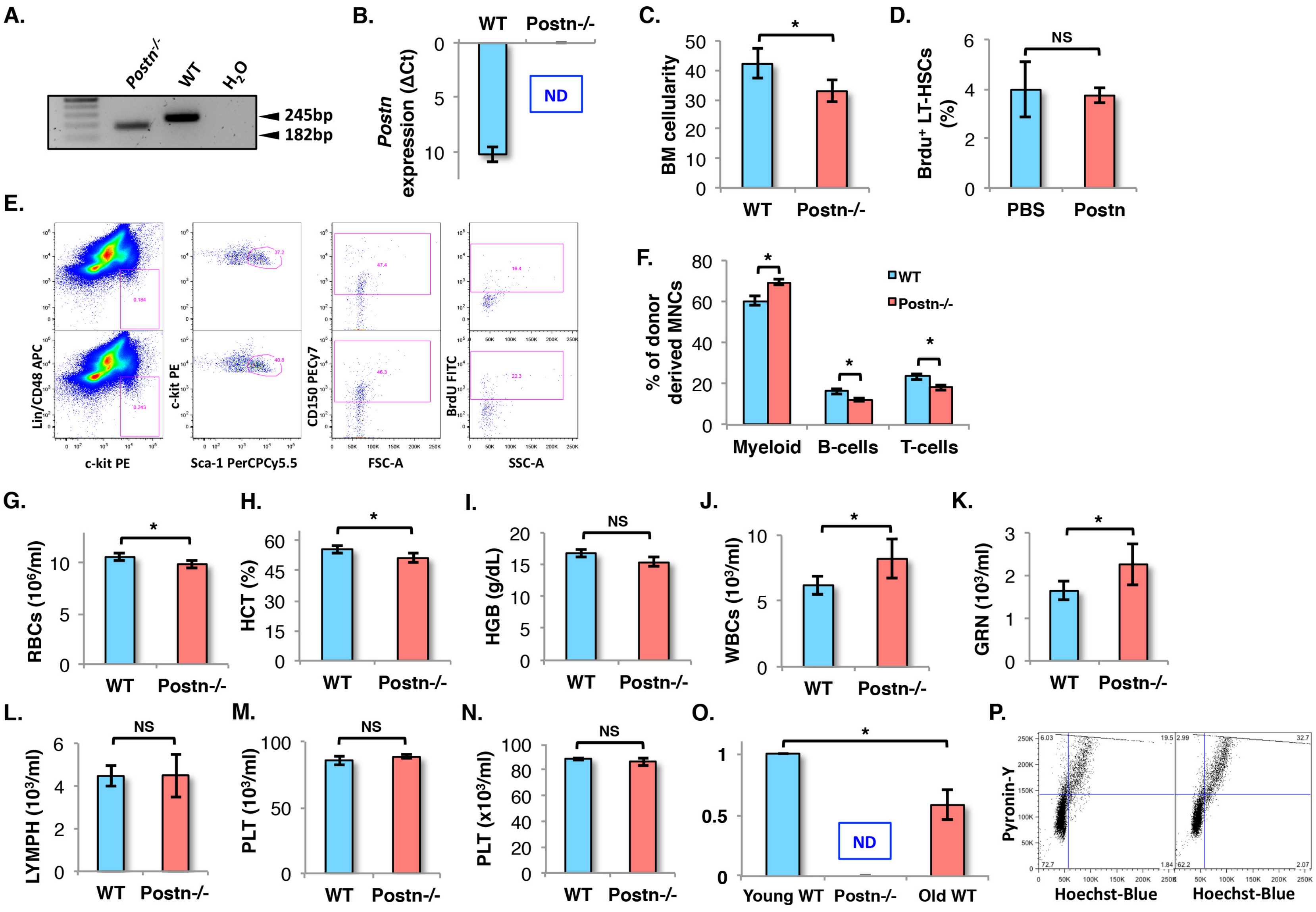

Supplementary Figure 2. Postn deficiency leads to pre-mature exhaustion of hematopoietic system

(A) *Postn*$^{-/-}$ mice were bred and genotyping was performed using tail tip DNA, PCR was performed using specific primers.

(B) Quantification of *Postn* expression in total BM cells from WT/*Postn*$^{-/-}$ mice by qRT-PCR. ΔCt values were plotted.

(C) Total BM mononuclear cells in hind limb bones of WT/*Postn*$^{-/-}$ mice. Total cell numbers in millions was plotted.

(D) BrdU incorporation assays to examine the proliferation status of HSCs in WT and *Postn*$^{-/-}$ mice. BrdU staining in addition to HSC markers in BM cells following 3 days of BrdU infusion (n=3, N=9, t test: NS p>0.05).

(E) Gating strategy to assess BrdU incorporation in LT-HSCs (identified as CD150^{+}CD48^{-} KLS cells) following 7 days pulse of BrdU to identify proliferating HSCs.

(F) Multi-lineage engraftment analysis for peripheral blood in secondary recipients that received total BM cells from WT/*Postn*$^{-/-}$ mice.

(G-M) Blood obtained from 8 week-old wild-type (WT) and *Postn*$^{-/-}$ mice was assessed for RBC count (E), hematocrit (F), hemoglobin level (G), WBC count (H), granulocytes (I), lymphocyte (J), and platelets (K) numbers.

(N) Platelet counts in peripheral blood of 16 week-old WT/*Postn*$^{-/-}$ mice.

(O) Quantification of *Postn* expression in total non-hematopoietic cells from BM of young (8 weeks) WT/*Postn*$^{-/-}$ and old (18 months) WT mice by qRT-PCR.

(P) Cell cycle analysis of the donor derived HSCs in the BM of secondary recipients. KLS cells identified within the donor derived (CD45.1^{+}) population were plotted for Hoechst and Pyronin Y label.

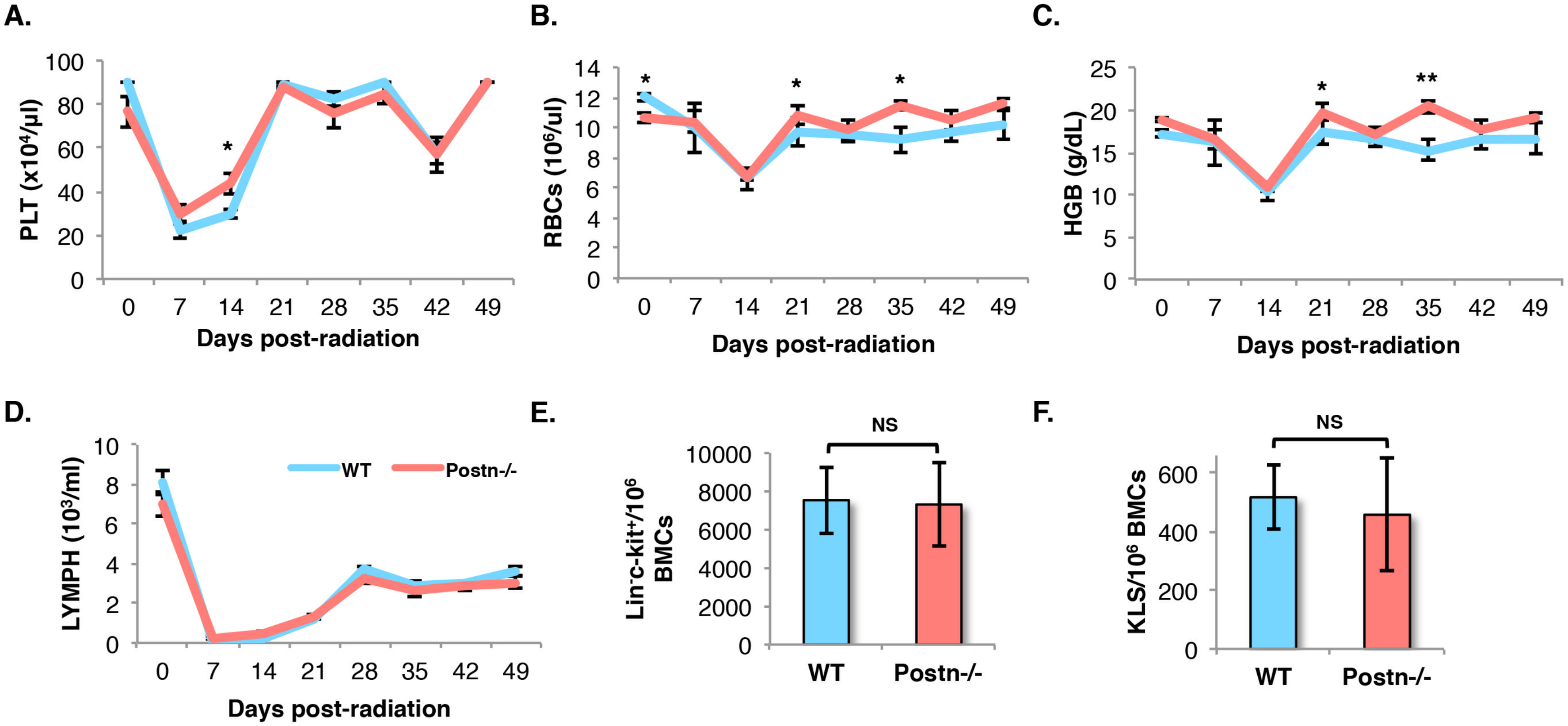

Supplementary Figure 3. Faster exhaustion of HSCs in *Postn*$^{-/-}$ mice following hematopoietic injury

(A-D) Following sub-lethal irradiation PB counts were measured weekly for 7 weeks. Platelet count (A), RBC count (B), HGB levels (C), and lymphocytes (D) were compared between FVB/NJ (WT) and *Postn*$^{-/-}$ (KO) mice (n=3, N=18, t test: ** $p<0.01$, * $p<0.05$).

(E,F) Flowcytometry based analysis performed on the BM cells of WT/*Postn*$^{-/-}$ mice after 8 weeks of sub-lethal irradiation to compare the number of lin$^-$c-kit$^+$ (E) and KLS (F) cells (n=3, N=9, t test NS $p>0.05$).

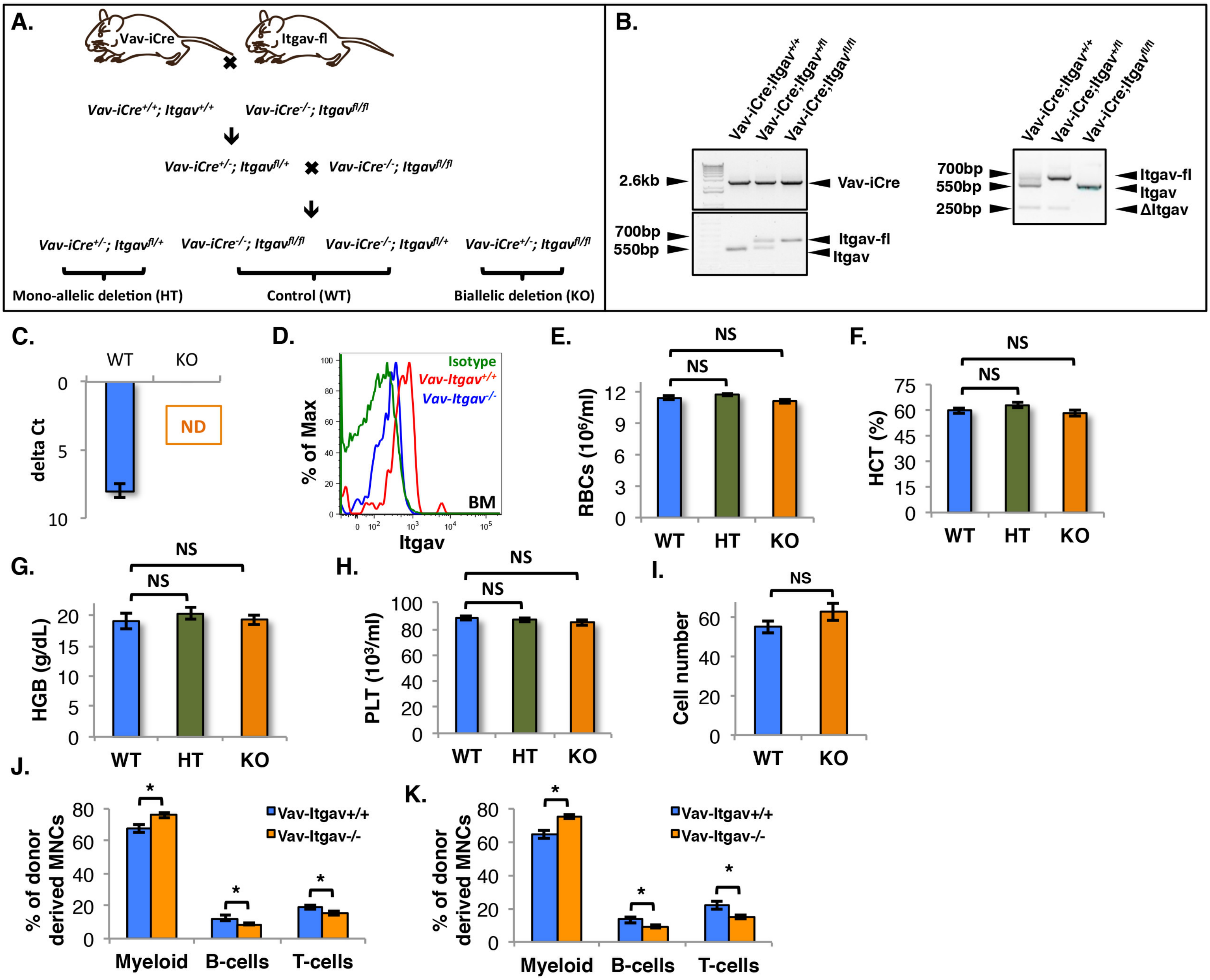

Supplementary Figure 4. ***Itgav$^{fl/fl}$;Vav-icre*** **mice show decreased proportion of BM HSCs**

(A) Schematic representation of breeding scheme used to generate *Vav-iCre^{+}; Itgav$^{+/+}$* (WT), *Vav-iCre^{+}; Itgav$^{+/-}$* (HT) and *Vav-iCre^{+}; Itgav$^{-/-}$* (KO) mice.

(B) Genotyping PCR performed on tail-tip (left) and BM (right) samples, to identify WT, HT and KO mice.

(C) BM cells from WT and KO mice were used to confirm lack of *Itgav* gene expression by qRT-PCR.

(D) BM derived mononuclear cells from WT and KO mice were used to confirm lack of Itgav protein expression in KLS cells by flowcytometry.

(E-H) Peripheral blood from 8 week-old littermate WT, HT and KO mice was analyzed for RBC counts (E), HCT values (F), HCT values (G) and platelet counts (H).

(I) Total BM mononuclear cells in hind limb bones of *Vav-Itgav$^{+/+}$* (WT)/*Vav-Itgav$^{-/-}$* (KO) mice. Total cell numbers in millions was plotted.

(J,K) Multi-lineage engraftment analysis in peripheral blood in secondary recipients that received total BM (J) or sorted KLS (K) cells from *Vav-Itgav$^{+/+}$* (WT)/*Vav-Itgav$^{-/-}$* (KO) mice (n=3, N=12, ttest: * p<0.05, Error bar represents SEM).

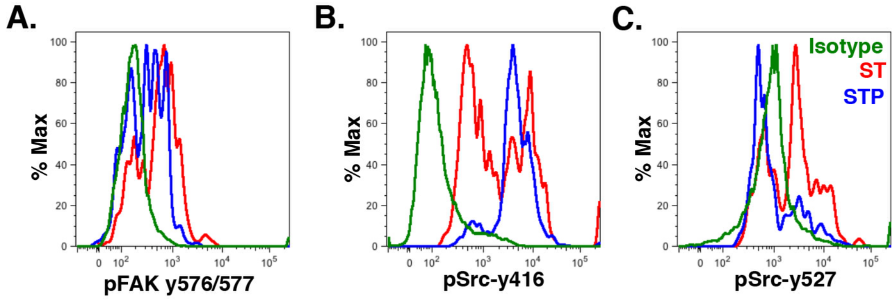

Supplementary Figure 5. ***Itgav*** **deficient HSPCs proliferate faster without any change in homing potential**

(A-C) Lineage depleted BM cells cultured for 2 days in the presence of SCF and TPO in serum free medium with/without Postn. After 2 days of culture the cells were harvested and stained for cell surface markers to identify HSCs in which the phosphorylation status of FAK (y576/577) (A), Src (y416) (B) and Src (y527) (C) was examined by flow cytometry.

Supplementary table 1: Sequences of the primers used

Primer name	Primer sequence (5'-3')
Mm β-actin	(F) GCTTCTTTGCAGCTCCTTCGT<br>(R) ATCGTCATCCATGGCGAACT
Mm Postn	(F) AAGTTTGTTCGTGGCAGCAC<br>(R) CCTCCTGTGGAAATCCTGGT
Mm CyclinD1	(F) TCCTGCTACCGCACAACGCA<br>(R) GGCGCAGGCTTGACTCCAGA
Mm CyclinD2	(F) CAGCTCCTGGGTGCAGTGTGC<br>(R) TTTGCTGGGGCAGCTTGCGA
Mm CyclinE1	(F) GCTCCGACCTTTCAGTCCGCT<br>(R) GACGGGAAGTGGGGAGGCTCT
Mm CyclinA1	(F) AGCTTGGCCAGGATCCCCCA<br>(R) TGCCCTCTTTCCCCGAGCAGG
Mm CyclinA2	(F) TCAGTAAACAGCCTGCCTTCACCA<br>(R) ACCTCCATTTCCCTAAGGTACGTGT
Mm p16Ink4a	(F) GTCGCAGGTTCTTGGTCACT<br>(R) TCTGCACCGTAGTTGAGCAG
Mm p19Ink4d	(F) TCCTGACGCCCTGAACCGCT<br>(R) TGTCCAGGAACCCGGTGCGA
Mm p15Ink4b	(F) GGGGCAAGTGGAGACGGTGC<br>(R) GAGCTGCGTCGTGCACAGGT
Mm p57kip2	(F) GAGTGCGCTGTGCTCGAGGG<br>(R) TCCATCGCTGTTCTGCTGGCTG
Mm p21cip1	(F) CAGGCGCAGATCCACAGCGAT<br>(R) GGGCAGCCCTAGGCTCCGAA
Mm p27kip1	(F) GCACTGTGGAGCAGACGCCC<br>(R) TGCGCAATGCTACATCCAATGCT
Mm Cdk2	(F) ACCCTGTGGTACCGAGCACCT<br>(R) GCACAGCGGGCAGAGACTGT
Mm Cdk4	(F) TGGACATGTGGAGCGTTGGCT<br>(R) AGTGCTGCAGGGCTCGGAAGG
Mm Cdk6	(F) GTTCCAGAGCCCGGCGTACC<br>(R) CCACAGCGTGACGACCACCG
Genotyping Postn mice	(F) GGT GCT TCT GTA AGG CCA TC<br>(R) GTG AGC CAG GAC CTT GTC ATA<br>(Int-as) AGC ACT GAC TGC GTT AGC AA
Genotyping Itgav mice	(F) GGTGACTCAATCTGTGACCTTCAGC<br>(R) CACAAATCAAGGATGACCAAACTGAG
Genotyping Vav-iCre mice	(F) CCATGGCACCCAAGAAGAAG<br>(R) GCTTAGTTTTCCTGCAGCGG
